# Supplementary material for: Impact of miRNA-mRNA Profiling and Their Correlation on Medulloblastoma Tumorigenesis
Source: Mol Ther Nucleic Acids. 2018 Jun 19;12:490–503. doi: 10.1016/j.omtn.2018.06.004 (PMC6070673; doi:10.1016/j.omtn.2018.06.004)
Supplement: Document S1. Figures S1–S3 and Table S5 [file mmc1.pdf]

OMTN, Volume *12*

Supplemental Information

Impact of miRNA-mRNA Profiling and Their Correlation on Medulloblastoma Tumorigenesis

Vinod Kumar, Virender Kumar, Amit Kumar Chaudhary, Donald W. Coulter, Timothy McGuire, and Ram I. Mahato

Supplemental information

Supplemental figures

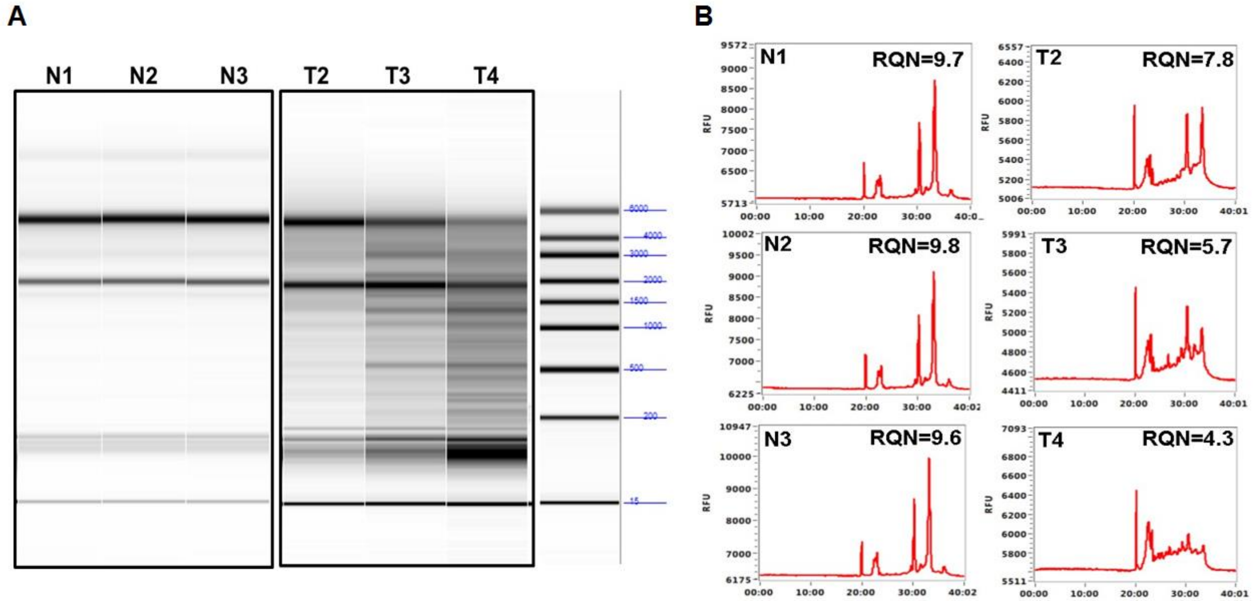

Figure S1. Electropherogram detailing of the RNA sample. (**A**) Gel image of total RNA samples, analyzed on the Agilent 2100 Bioanalyzer System using the Eukaryote Total RNA Nano assay. (**B**) The RNA quality score (RQN) was used to test the intactness and quality of RNA samples used in the RNA-Seq analysis.

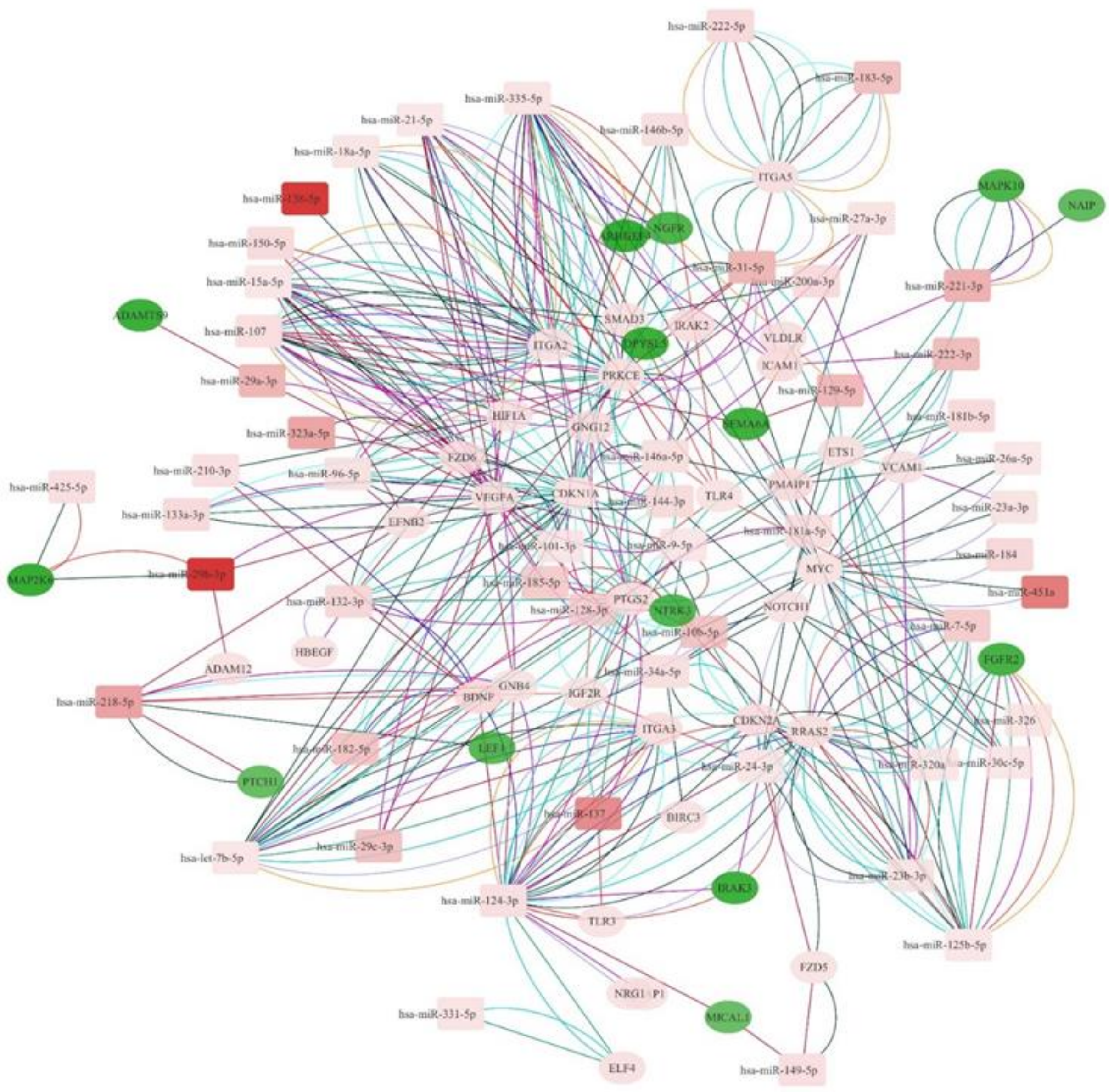

Figure S2. Molecular network identified by IPA between miRNA and mRNA in MB.

The rectangle represents the miRNAs and circles mRNAs.

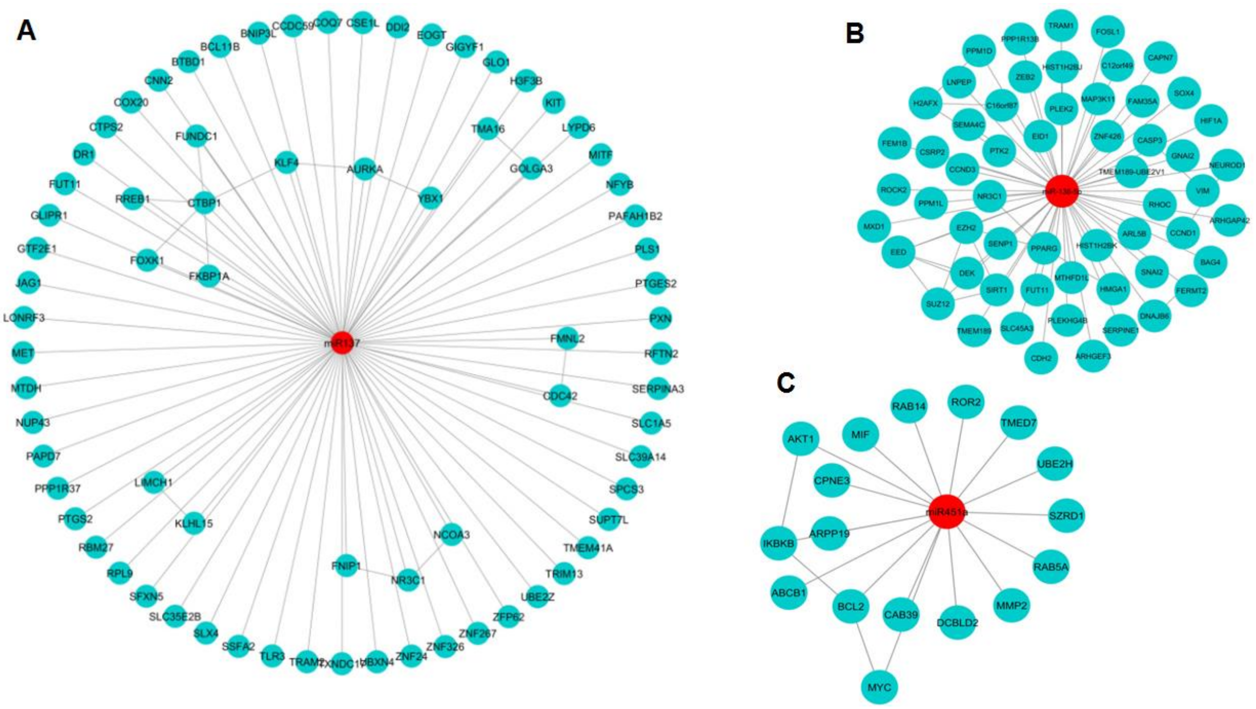

Figure S3. IPA of the most deregulated miRNAs and their target genes. (A). miR-137. **(B).** miR-138-5p. **(C).** miR-451a.

Description of Supplementary Tables

Supplementary Table 1S. Comparison of gene expression profile in medulloblastoma and normal cerebellar tissues obtain from RNA-Seq.

Supplementary Table 2S. The number of genes and canonical pathways specific to the cerebellum. The log fold change five or above was considered as significant.

Supplementary Table 3S. The number of genes involved in various diseases and pathways specific to the cerebellum.

Supplementary Table 4S. Top diseases and functions controlled by the genes which express in the cerebellum. The log fold change five or above was considered as significant.

Supplementary Table 5S.

(A) Clinical data and molecular sub-type of primary MB specimens utilized in this investigation

(B) The top five cellular function and top upstream regulators and their predicted activation from IPA in MB specimens

Supplementary Table 6S. The mRNA and miRNA target correlation. The red color depicts the positive correlation and green color negative correlation.

Supplementary Table 5 S.

(A) Clinical data and molecular sub-type of primary MB specimens utilized in this investigation.

**Variables**	
Sample size	10
Age (years)	Median (20.8)
	Range (8-46)
Gender	Male (4)
	Female (6)
Desmoplasia	2
Tumor grade	WHO grade IV (4)
	WHO grade II (1)
Metastasis state	No

(B) The top five cellular function and top upstream regulators and their predicted activation from IPA in MB specimens.

**Top molecular and cellular functions**		
**Molecular and cellular functions**	**Molecules**	**p-value**
Cellular movement	157	1.83E-04 – 7.45E-22
Cell death and survival	205	1.85E-04 – 5.86E-19
Cellular development	202	1.85E-04 – 3.70E-14
Cellular growth and proliferation	201	1.85E-04 – 3.70E-14
Cell morphology	95	1.62E-04 – 2.98E-13
**Top upstream regulators**		
**Upstream regulators**	**Predicted activation**	**p-values**
TNF	Inhibited	1.36E-14
SP1	Inhibited	4.36E-11
SMARCA4	Inhibited	1.07E-09
Cg	Inhibited	4.57E-09
ATF4	Inhibited	5.51E-09
