# Supplementary material for: Impact of miRNA-mRNA Profiling and Their Correlation on Medulloblastoma Tumorigenesis
Source: Mol Ther Nucleic Acids. 2018 Jun 19;12:490–503. doi: 10.1016/j.omtn.2018.06.004 (PMC6070673; doi:10.1016/j.omtn.2018.06.004)
Supplement: Document S2. Article plus Supplemental Information [file mmc7.pdf]

Molecular Therapy
Nucleic Acids
Original Article

Impact of miRNA-mRNA Profiling and Their Correlation on Medulloblastoma Tumorigenesis

Vinod Kumar,[1] Virender Kumar,[1] Amit Kumar Chaudhary,[1] Donald W. Coulter,[2] Timothy McGuire,[3] and Ram I. Mahato[1]

[1]Department of Pharmaceutical Sciences, University of Nebraska Medical Center, Omaha, NE 68198, USA; [2]Department of Pediatrics, University of Nebraska Medical Center, Omaha, NE 68198, USA; [3]Department of Pharmacy Practice, University of Nebraska Medical Center, Omaha, NE 68198, USA

Medulloblastoma (MB) is a clinically challenging, childhood brain tumor with a diverse genetic makeup and differential miRNA profile. Aiming to identify deregulated miRNAs in MB, the miRNA expression profile of human MB samples was compared to that of normal cerebellar tissues. As a result, 8 upregulated and 64 downregulated miRNAs were identified in MB samples. Although various algorithms have been developed to predict the interaction between miRNA-mRNA pairs, the complexity and fidelity of miRNA-mRNA remain a concern. Therefore, to identify the signatures of miRNA-mRNA interactions essential for MB pathogenesis, miRNA profiling, RNA sequencing, and ingenuity pathway analysis (IPA) were performed in the same primary human MB samples. Further, when miR-217 was inhibited, a significant upregulation of predicted target genes SIRT1, ROBO1, FOXO3, and SMAD7 in HDMB03 cells was observed, confirming the validity of our approach. Functional analysis revealed that the inhibition of miR-217 in HDMB03 cells suppresses colony formation, migration, invasion, promoted apoptosis, and arrested cell population in S phase, indicating that manipulation of miR-217 may have a therapeutic potential for MB patients. Therefore, our study provides an essential platform for future investigations of specific miRNAs responsible for MB pathogenesis.

INTRODUCTION

Medulloblastoma (MB) is the most common malignant brain tumor in children, mainly arising in the cerebellum.[1,2] MB is classified into four subgroups: wingless (Wnt) group, sonic hedgehog (Shh) group, group 3, and group 4.[3–5] Each subgroup has different origins and pathogenesis and differs not only in genetic signature but also in response to clinical therapy. Beside these classifications, Schwalbe et al.[6] further classified MB into seven subgroups, and Cavalli et al.[7] further classified MB into 12 subgroups. The recognition of an increasing number of subgroups in MB suggests that unique molecular subgroups may arise from a different group of clones.[8–10] Based on histopathological rather than molecular characteristics, the World Health Organization (WHO) classified MB into five subtypes: classic, desmoplastic or nodular, MB with extensive nodularity, anaplastic, and large-cell MB.[1,11,12] Treatment of MB remains a challenge due to high tumor heterogeneity, chemoresistance, poor permeability across the blood-brain barrier (BBB), and microRNA (miRNA) dysregulation. The MB tumor has different genetic signatures and miRNA expression profiles compared to normal cerebellar tissue. It is important to understand the genetic, epigenetic, and translational programs that drive MB tumorigenesis and can lead to the development of novel therapeutics.

miRNAs are short, non-coding endogenous RNAs that regulate many biological processes through gene regulation.[13,14] They may bind to complementary sequences in the 3′ UTR of target genes[15] and alter the expression by repressing translation and/or inducing deadenylation and subsequent mRNA degradation.[13,16–18] Single miRNAs can target up to several hundreds of mRNAs, and a single mRNA can be targeted by many miRNAs, which enables them to regulate many signaling pathways and cellular processes.[19,20] Their altered expression is associated with many types of cancers, including MB. miRNAs affect the expression of tumor suppressor genes, oncogenes, and other signaling molecules in MB.[10]

It is important to analyze key miRNAs and miRNA-mRNA interactions before the clinical development of miRNA-based therapeutics. Various algorithms predicting miRNA targets rely on sequence complementarity, evolutionary conservation, and thermodynamic stability, but false interpretations remain a problem, because a substantial fraction of these interactions may depend on cell type and binding of the additional cofactors. On the other hand, miRNA target prediction can be improved by using the inverse correlation between overexpressed miRNAs and downregulated genes from the same tumor samples, or vice versa.[21] RNA sequencing (RNA-seq) has emerged as a revolutionary tool for transcriptomics with high-throughput at an incomparable level of accuracy and sensitivity.[22,23] Compared to microarray analysis, which has limited sensitivity and is prone to cross-hybridization

Received 20 April 2018; accepted 2 June 2018;
https://doi.org/10.1016/j.omtn.2018.06.004.

Correspondence: Ram I. Mahato, PhD, Department of Pharmaceutical Sciences, University of Nebraska Medical Center (UNMC), 986025 Nebraska Medical Center, Omaha, NE 68198-6025, USA.
E-mail: ram.mahato@unmc.edu

 © 2018 The Authors.
This is an open access article under the CC BY license (http://creativecommons.org/licenses/by/4.0/).

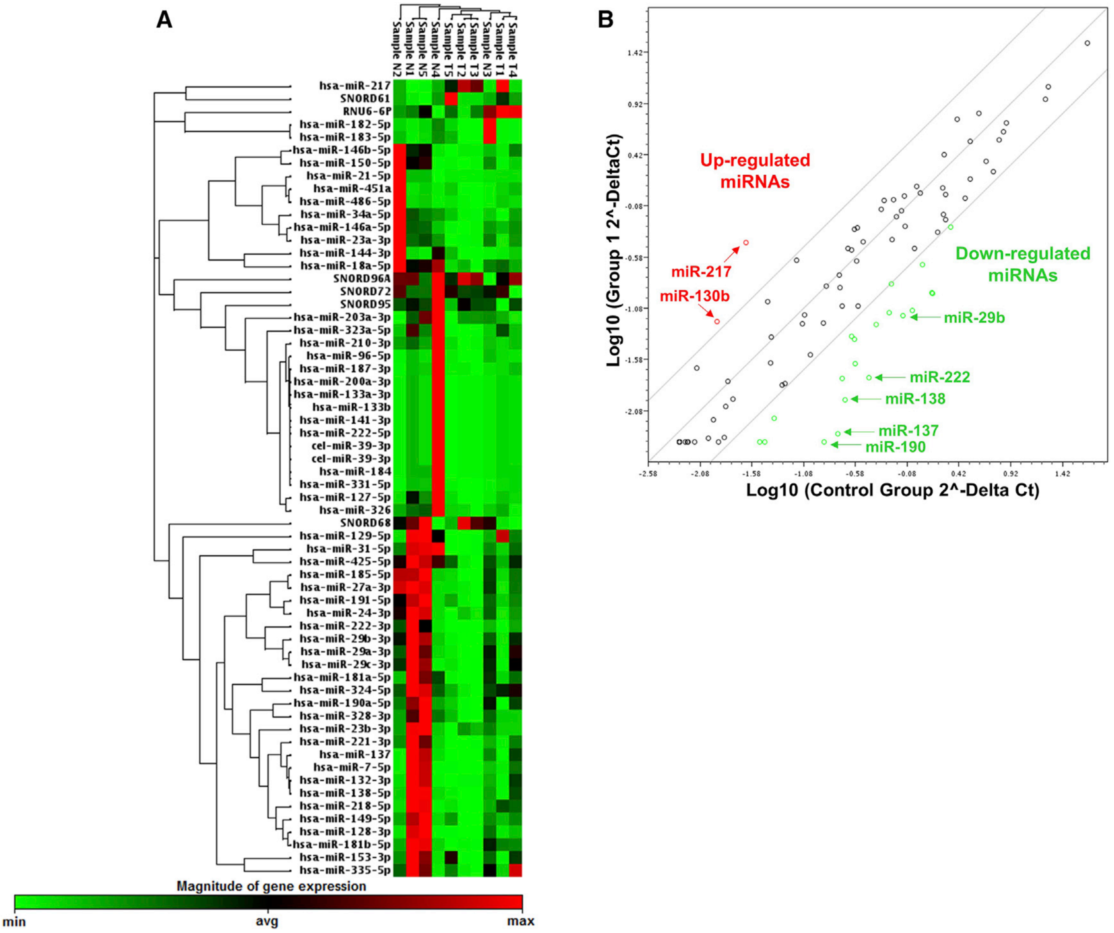

Figure 1. miRNA Expression Pattern in Different Human MB and Normal Cerebellar Tissues

(A) Heatmap analysis of miRNAs in normal and MB tissues. Unsupervised hierarchical clustering analysis was performed for significantly (>2-fold change) expressed miRNAs. miRNAs are indicated in columns, and tumor samples are indicated in rows. N1, N2, N3, N4, and N5 are the normal cerebellar tissue samples; and T1, T2, T3, T4, and T5 are the MB samples. A green-to-red color scale shows an increase in normalized miRNA expression on a log scale. (B) Scatterplot of miRNAs significantly upregulated (y axis) and downregulated (x axis) in MB tissues compared to normal cerebellar tissues. The scatterplot graph was generated by averaging $\log_2(2^{-\Delta\Delta\ Ct})$ values from real-time PCR analysis. A fold change of two or more was considered significant. Each circle corresponds to one miRNA.

between homologous DNA fragments, RNA-seq has higher sensitivity and ability to detect somatic mutations and splicing isoforms.[22,24] Earlier studies using RNA-seq in MB were mostly conducted in cell lines and mouse tissues with improper tissue control.[25,26] However, very few studies have shown the miRNA and mRNA expression patterns with their correlation in the same human MB patient.[27] To fill this gap, in this study, we performed RNA-seq and miRNA profiling to study differential gene expression pattern in human MB patient tissues. We also examined the miRNA-mRNA correlation by ingenuity pathway analysis (IPA). We identified several differentially expressed genes, miRNAs, miRNA-mRNA correlations, and canonical pathways that may lead to actionable targets in MB. We found that miR-217 was upregulated in human MB patient tissues and cell lines. Inhibition of miR-217 induced apoptosis and reduced migration, invasion, and colony formation in HDMB03 cells.

RESULTS

Identification of Differentially Expressed miRNAs and Hierarchical Clustering Analysis

miRNA profiles were generated for primary human MB specimens using qRT-PCR. Five normal cerebellar and five MB tissues from different patients were profiled using a QIAGEN 96-well plate (MIHS-108ZF). This plate contains 84 miRNAs known to play important roles in brain cancer along with six normalization controls (SNORD61, SNORD68, SNORD72, SNORD95, SNORD96A, and RNU6B/RNU6-2). The miRNA expression profiles obtained from the normal and MB samples were clustered by unsupervised hierarchical clustering analysis and generated a heatmap dendrogram. The heatmap dendrogram shows distinctive patterns and clear separation of control versus MB specimen (Figure 1A). The scatterplot, along with the heatmap dendrogram, shows that most of the miRNAs were downregulated in MB patient tumor samples (Figure 1B). We

Table 1. Summary of Differentially Expressed miRNAs in Primary MB Tissues compared to Normal Cerebellar Tissues

miRNA	Fold Change
Upregulated miRNA	
miR-130b-3p	2.75
miR-148a-3p	1.89
miR-15b-5p	2.37
miR-17-5p	1.766
miR-19b-3p	1.75
miR-216a-5p	2.53
miR-217	11.89
miR-25-3p	2.37
Downregulated miRNA: >10-Fold Change	
miR-10b-5p	−24.92
miR-128-3p	−17.79
miR-129-5p	−33.51
miR-132-3p	−12.26
miR-133a-3p	−11.02
miR-133b	−11.02
miR-137	−64.00
miR-138-5p	−121.33
miR-141-3p	−11.02
miR-144-3p	−10.03
miR-153-3p	−13.88
miR-181b-5p	−11.09
miR-182-5p	−24.91
miR-183-5p	−27.91
miR-184	−13.09
miR-185-5p	−19.90
miR-190a-5p	−20.78
miR-200a-3p	−10.87
miR-218-5p	−49.39
miR-221-3p	−38.62
miR-222-3p	−31.02
miR-222-5p	−11.02
miR-29a-3p	−36.12
miR-29b-3p	−123.30
miR-29c-3p	−26.92
miR-31-5p	−35.57
miR-323a-5p	−46.89
miR-451a	−74.32
miR-7-5p	−20.47

observed that 8 miRNAs out of 84 were significantly upregulated, and 64 were downregulated in MB specimens (Table 1). Statistical analysis was carried out to investigate the fold change in the expression of individual miRNAs. Among the upregulated miRNAs, miR-217 was overexpressed compared to normal cerebellar tissues by 11.8-fold (Figure 2A). The other significantly upregulated miRNAs are miR-130b (2.7-fold), miR-148a (1.8-fold), miR-15b (2.3-fold), miR-17 (1.7-fold), miR-19b (1.7-fold), miR-216a (2.5-fold), and miR-25 (2.3-fold) (Figure 2A). Among the downregulated miRNAs, some of the most significant miRNAs are miR-29b (−123.3-fold), miR-138 (−121.3-fold), miR-451a (−74.3-fold), miR-137 (−60-fold), miR-323a (−46.8-fold), and miR-218 (−49.3-fold) (Figure 2B).

miR-217 in MB Cell Lines

We decided to investigate miR-217 expression further, because miR-217 has been reported to be strongly involved in many cancers, as it plays a critical role in cell survival and proliferation, but its role in MB is elusive.[28–32] To increase the clinical relevance of miRNA profiling in human MB patient tissues where miR-217 expression was highly upregulated, we used two different controls to validate our finding from human patient tissues. To confirm miR-217 expression in Daoy, HDMB03, and D341 MB cell lines, we used neuronal progenitor cells (NPCs) and SVG p12 as a control cell line and measured miRNA expression by qRT-PCR. miR-217 expression was significantly upregulated in all three MB cell lines (Daoy, HDMB03, and D341) compared to the control NPCs and SVG p12 cells (Figures 2C and 2D). The maximum upregulation of miR-217 was observed in the HDMB03 cell line when compared with NPCs and SVG p12 cell lines by 1,448.6 and 5,103.2 folds, respectively. Since human patient MB and normal cerebellar tissues were used in miRNA profiling, and the same expression pattern of miR-217 was observed in various MB cell lines, these findings can be used as validation for future *in vivo* studies.

Statistical Analysis for RNA-Seq

RNA-seq was carried out in six samples (three MB patients and three normal human patient tissues). The differential gene expression analysis between the control and MB patient tissues was calculated by using Spliced Transcripts Alignment to a Reference (STAR) and RNA-Seq by Expectation Maximization (RSEM) annotation.[33–35] The normalized expression (Student's t test) abundance of all available genes in TPM (transcript per kilobase million) values were calculated (Table S1). The TPM values were calculated as follows: (1) the read counts were divided by the length of each gene in kilobases (reads per kilobase: RPKs); (2) all the RPK values in a sample were counted and divided by 1,000,000 ("per million" scaling factor); and (3) dividing the RPK values by the "per million" scaling factor gives the TPM values. The significance level was determined by multiple testing of the adjusted p values (q value) $\leq$ 0.05 for all significant genes and sorted according to the fold change (MB versus control) from the smallest to the largest (Table S1). Statistical alignment indicated that the data were uniform (no outliers concerning alignment proficiency) and of high quality and provided adequate sequencing depth to perform differential expression testing between normal and disease groups.

Identification of Differentially Expressed Genes

To select differentially expressed genes (DEGs), we ranked genes by the $\log_2$ fold change with adjusted p values $\leq$ 0.05. We identified

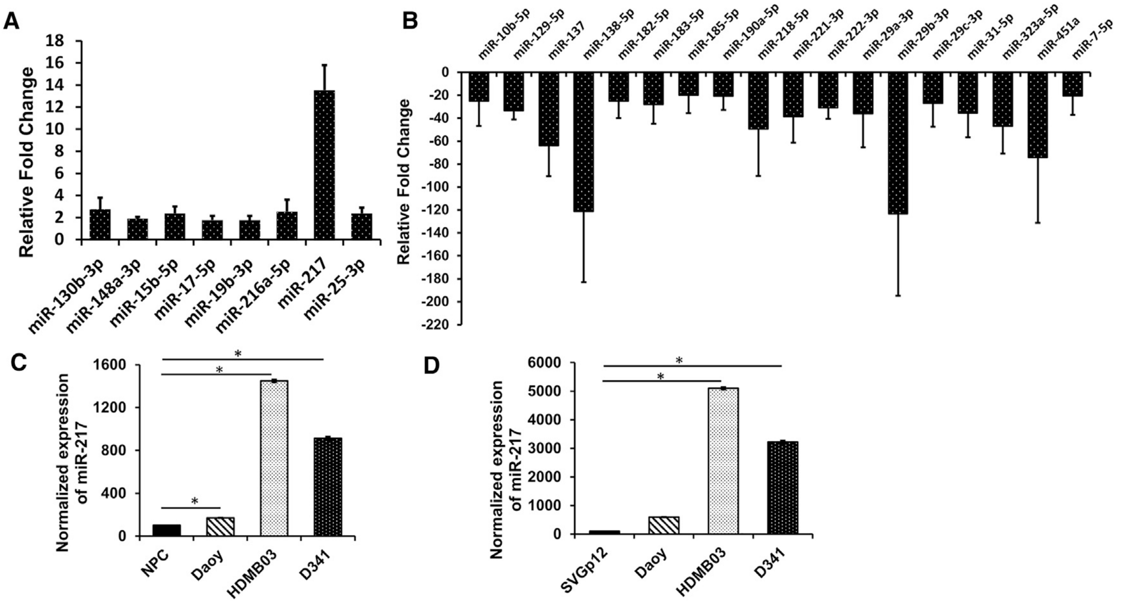

Figure 2. miRNA Profiling of MB and Normal Cerebellar Tissues

(A) Upregulated miRNA scripts. (B) Downregulated miRNA scripts (>20-fold change). In both cases, relative fold change with respect to normal cerebellar tissues was used to calculate the final fold change. The housekeeping genes SNORD61, SNORD68, SNORD72, SNORD95, SNORD96A, and RNU6B/RNU6-2 were used as internal controls. Expression of miR-217 in different MB cell lines by real-time qPCR compared to (C) neuronal progenitor cells (NPC) and (D) SVG p12. Data are shown as the mean ± SD of three separate experiments (Student's t test; *p < 0.05; n = 3).

57,773 DEGs, in which 12,087 genes were significantly deregulated. We identified that 7,923 genes were downregulated, and 4,164 were upregulated (Figure 3; Table S1). Among the overexpressed genes, PTGDS (13.761-fold) had the highest $\log_2$ fold change in tumor versus non-tumor tissues, followed by TNFRSF18 (13.387-fold), glial fibrillary acidic protein (GFAP) (12.296-fold), BARHL1 (12.175-fold), and KIF26A (11.719-fold). Among the downregulated genes, GREM1 (−11.652-fold) had the maximum fold change, followed by INHBA (−11.341-fold), DSC3 (−11.209-fold), IL1B (−11.209-fold), and IGFN1 (−10.890-fold) (Table 2).

IPA

The IPA was performed for the genes that express in the brain. The list of brain-specific genes was obtained from the Human Protein Atlas (http://www.proteinatlas.org), which contains 12,936 genes. The RNA-seq results reveal that 8,038 genes out of 12,074 were brain enriched according to the Human Protein Atlas. We further confirmed that our RNA-seq results from the medulloblastoma OMIM database. The four genes from the OMIM database—PTCH2, CTNNB1, SUFU, and BRCA2—were also significant in our RNA-seq analysis (adjusted p value $\leq$ 0.05). These findings further validated our results.

IPA of DEGs revealed 381 significant canonical pathways (Table S2) and 25 significant molecular and cellular functions in various diseases (Table 3; Table S3). The top five canonical pathways are axon guidance signaling, hepatic fibrosis due to hepatic stellate cell (HSC) activation, the STAT3 pathway, signaling by Rho family GTPases, and the phosphate and tensin homolog (PTEN) signaling pathway (Table 3). The major contributing genes to the top five pathways are listed in Table 3. The top five significant cellular functions that are represented by DEGs are cell movement, cell death, cell survival, cellular proliferation, and cell morphology (Table 3; Table S4). Most of these pathways are associated with nervous system development and neuronal migration. The top upstream regulators, which were inhibited in IPA, includes TNF, SP1, SMARCA4, and ATF4 (Table S5).

Integrative Analysis of miRNA-mRNA Correlation Reveals Several Regulatory Networks in MB

In an effort to verify that the targets of differentially expressed miRNAs do regulate the mRNA expression levels in MB, we performed miRNA and mRNA integrated analysis (MMIA) (Figure 4; Figure S2). We first identified the miRNA targets using the miRTarBase database. The resulting miRNA targets were further filtered to the genes that are differentially expressed in the cerebellum based on RNA-seq analysis. These target genes were used to perform IPA. The results of MMIA revealed several genes targeted by one miRNA. We established a correlation value between the upregulated miRNAs and their downregulated target genes from the miRNA profiling and RNA-seq data (Table S6). To address the functional significance of MMIA, the IPA was performed focusing on deregulated mRNA targets for both up- and downregulated miRNAs in MB patient tissues. Of particular interest, upregulated miR-217 was predicted to target SIRT1, FOXO3, ROBO1, and SMAD7. The IPA was performed for some of the most significant downregulated miRNAs, such as miR-15b, miR-137, miR-138-5p, miR-451a, and

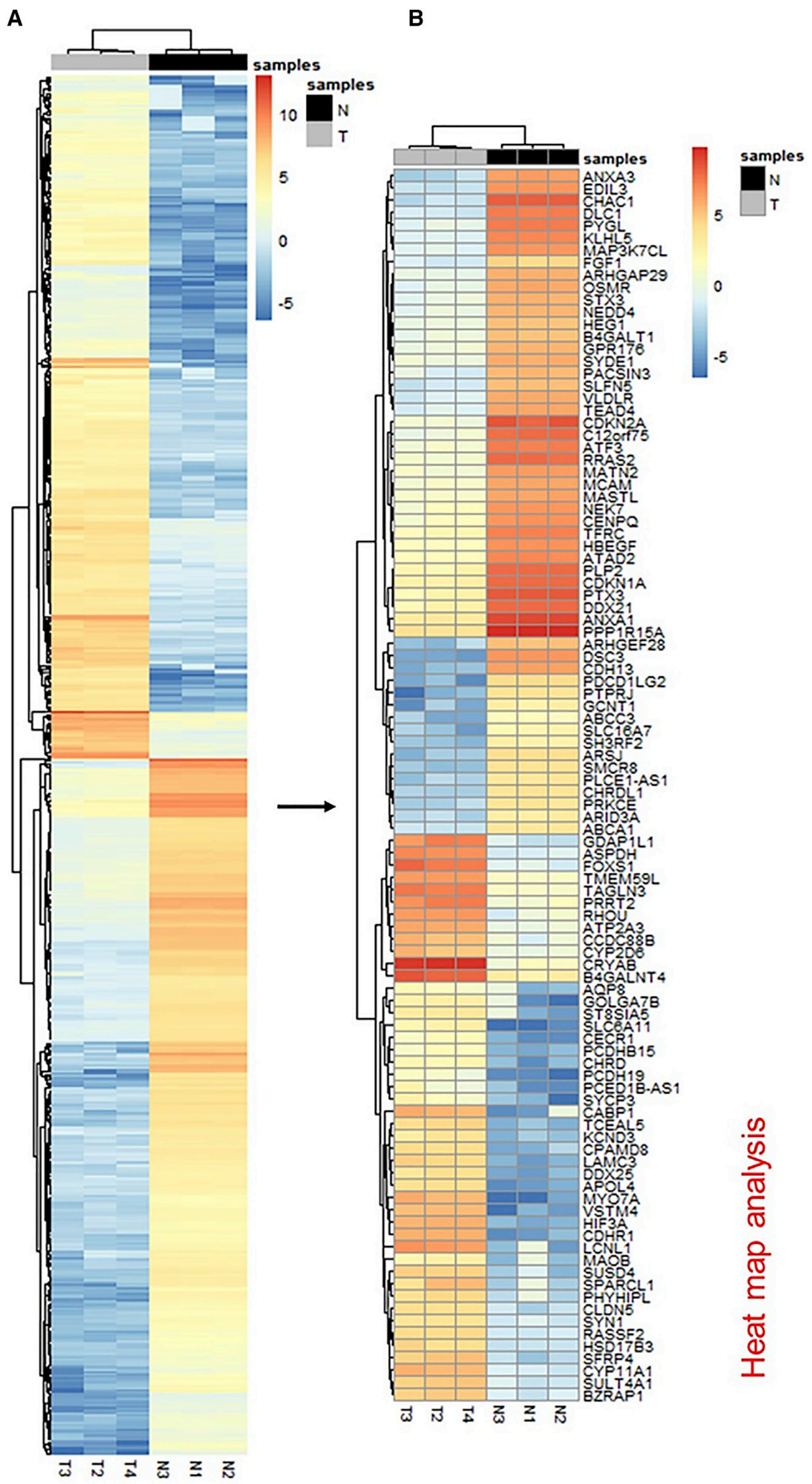

Figure 3. Analysis of the Differentially Expressed Genes in MB Patient Tissue

(A) A heatmap of 675 differentially expressed genes; (B) a heatmap of selected 100 differentially expressed genes and their log-normalized expression in normal (N; black) and MB (T; gray) samples were drawn. Red represents upregulation, and blue represents downregulation.

Table 2. Top Differentially Expressed Genes with Log_2 Fold Change and p Values

Symbol	Gene Name	Log_2 (Fold Change)	p Value
Upregulated			
MYO7A	myosin VIIA	11.112	7.89E−05
LCNL1	lipocalin-like 1	11.216	3.4E−03
AMER2	APC membrane recruitment protein 2	11.384	1.3E−03
DUSP26	dual specificity phosphatase 26	11.391	4.5E−03
CABP1	calcium-binding protein 1	11.406	8.8E−04
KIF26A	kinesin family member 26A	11.719	6.44E−05
BARHL1	BarH-like homeobox 1	12.175	5.30E-05
GFAP	glial fibrillary acidic protein	12.296	8.63E−05
TNFRSF18	TNF receptor superfamily member 18	13.387	2.4E−03
PTGDS	prostaglandin D2 synthase	13.761	1.43E−05
Downregulated			
GREM1	gremlin 1, DAN family BMP antagonist	−11.652	3.5E−04
INHBA	inhibin beta A subunit	−11.341	7.08E−05
DSC3	desmocollin 3	−11.209	1.48E−07
IL1B	interleukin-1 beta	−11.196	8.10E−05
IGFN1	immunoglobulin-like and fibronectin type III domain containing 1	−10.890	8.30E−05
ALPK2	alpha kinase 2	−10.818	2.37E−05
GDF15	growth differentiation factor 15	−10.708	3.72E−05
STC2	stanniocalcin 2	−10.666	2.04E−07
FGF5	fibroblast growth factor 5	−10.663	3.68E−04
KRT18	keratin 18	−10.523	5.60E−07

TNF, tumor necrosis factor.

miR-29b (Figure 5; Figure S3). The target genes for the significantly deregulated miRNAs regulate both cell proliferation and the developmental signaling pathway. Also, this approach will help in minimizing the false-positive target for particular miRNAs and mRNAs.

Validation of miR-217 Targets in MB

To evaluate the integration of miRNA and mRNA analysis, evaluate the predicted target genes, and gain the potential role of differentially expressed miRNAs in MB, we first tested the most significantly upregulated miRNA, miR-217. Several putative miR-217 targets predicted by TargetScan and our IPA were downregulated in an MB specimen compared to normal cerebellar tissue (Figure 5A). These include the genes with an established role in cell cycle and response to DNA damage (SIRT1 and SMAD7), axon guidance and neuronal precursor cell migration (ROBO1), and FOXO3 transcription factor. To assess whether the miRNA target genes obtained from IPA had an inverse expression to miR-217 level in MB, the expression levels of SIRT1, SMAD7, ROBO1, and FOXO3 gene were measured by qPCR in the HDMB03 cell line as well as in MB primary tumor by immunohistochemistry (IHC). A strong negative correlation was observed, since these genes were downregulated (Figure 6) and miR-217 was upregulated in the HDMB03 cell line and MB primary specimens (Figures 1, 2C, and 2D).

To address whether miR-217 regulates predicted target genes, the HDMB03 cell line was transiently transfected with antimiR-217 complexed with Lipofectamine. Significant upregulation of predicted targets SIRT1, SMAD7, ROBO1, and FOXO3 were observed at gene and protein levels, compared to scrambled antimiR-217 (Figures 6D and 6E). These results of miR-217 target genes and their proteins indicated that the MMIA was a useful approach for identification of miRNA-regulated genes in MB.

miR-217 Inhibition Increases Apoptosis and Cell Population in S Phase

To investigate the biologic function of miR-217 in MB, we performed apoptosis and cell-cycle analysis by flow cytometry. miR-217 was shown to be associated with cancer cell proliferation and cell-cycle transition.[30] The apoptotic rate was significantly higher (48.5%) when miR-217 was inhibited in HDMB03 cells compared to scrambled (32.5%) (Figure 7). Also, inhibition of miR-217 promoted more HDMB03 cells into S phase (52.5% in antimiR-217 and 45% in scrambled), accompanied with the decrease of G2 phase (14% in antimiR-217 and 19.78% in scrambled), compared to scrambled control (Figure 7). These results suggest that inhibiting miR-217 significantly inhibits the proliferation and induces the apoptosis of MB cells.

Table 3. Top Five Canonical Pathways and Differentially Expressed Genes in That Pathway with Their p Values and Percentage Overlap

Ingenuity Canonical Pathways	p Value	Genes	Overlap
Axonal guidance signaling	7.42E−06	MME, RAC2, PAPPA2, PLCB2, BDNF, SEMA6A, VEGFA, GNB4, EFNB2, MICAL1, ITGA3, NGFR, NTRK1, PRKCE, MMP11, SEMA4A, ROBO2, GNG12, ITGA2, PTCH1, TUBA4A, ITGA5, FGFR2, DPYSL5, ADAMTS9, SEMA3A, RRAS2, ADAM12, NTRK3, GNAO1, FZD6, FZD5, PRKCH, SEMA3C	7.7% (34/442)
Hepatic fibrosis/hepatic stellate cell activation	1.22E−05	VCAM1, ICAM1, MYH9, EDNRB, FGF2, SMAD3, FGFR2, BAMBI, COL8A1, IGFBP5, FGF1, VEGFA, COL1A2, TLR4, IGF2, EDN1, NGFR, ECE1, SERPINE1	10.5% (19/181)
STAT3 pathway	3.12E−05	MYC, GHR, RRAS2, NTRK3, NGFR, NTRK1, CDKN1A, MAPK10, FGFR2, TNFRSF11A, IGF2R	15.1% (11/73)
Signaling by rho family GTPases	3.28E−05	ARHGEF4, NEDD4, ITGA2, CDH6, ITGA5, CDC42EP3, FGFR2, CDH15, IQGAP1, DES, GNB4, ITGA3, CDH5, GNAO1, RHOU, MAPK10, CDC42EP1, GFAP, ACTG2, CDH13, ACTC1, GNG12	8.9% (22/246)
PTEN signaling	4.82E−05	RAC2, ITGA2, ITGA5, FGFR2, TNFRSF11A, IGF2R, SYNJ2, ITGA3, RRAS2, GHR, NTRK3, NGFR, CDKN1A, NTRK1	11.8% (14/119)

The percentage overlap value was calculated as follows: number of genes or proteins in the input mapped to the pathway divided by total number of genes or proteins known to be annotated to the term in the IPA knowledge base.

Inhibition of miR-217 Reduces Migration, Invasion, and Colony Formation

To demonstrate the role of miR-217 in the growth and metastasis of MB, we performed the migration, invasion, and clonogenic assay after transfecting HDMB03 cells with scrambled and miR-217 inhibitor. The cells transfected with antimiR-217 showed a reduction in cell migration by 63.6%, compared to the scrambled miRNA control (Figure 7). Consistent with these results, inhibition of miR-217 also showed a reduction in invasion of HDMB03 cells by 74.68%, compared to the scrambled miRNA control (Figure 7). miR-217 has been reported to promote cancer cell proliferation.[36,37] To evaluate the role of miR-217 in colony formation in the HDMB03 cell line, we transfected the cells with antimiR-217 and scrambled miRNA after complex formation with Lipofectamine RNAiMax. The cells were further grown for 12 days. The miR-217-transfected cells showed a reduction in colony formation by 62.5%, compared to scrambled miRNA control (Figure 7D). These results indicate that miR-217 exerts oncogenic characteristics in group 3 MB.

DISCUSSION

Despite tremendous clinical and biological heterogeneity, treatment of MB is relatively uniform and consists of surgery followed by craniospinal radiation and chemotherapy.[1] Cranial irradiation can be catastrophic in young children, causing severe cognitive impairment, whereas chemotherapy can also cause severe long-term complications. For MB patients younger than 3 years of age, treatments excluding radiation are used. Although the cure rate of MB patients has improved, the harsh and intense nature of treatments often leads to lifelong disabilities.[38,39] Thus, new, effective, and less toxic therapies are needed for MB treatment. Taking all these limitations into consideration, this study was performed to identify miRNA-mRNA pairs, which can inform the prognosis and serve as potential targets for improved therapeutic outcomes. The possibility of modulating the expression of dysregulated miRNAs has the potential for tumor suppression, but these phenomena have largely been un-exploited. Since miRNAs are single-stranded nucleotides involved in mRNA degradation and destabilization, they can be used as a therapeutic tool.[13,40] The first step in this experimental approach of molecular therapeutics requires profiling of miRNA and their mRNA targets in patient samples. Our miRNA profiling results are in good agreement with the work of Ferretti et al.,[41] who showed that most of the miRNAs were downregulated in MB tumor samples from patients when compared to matched normal tissues, only a few miRNAs were upregulated (Figure 1).

Since a single miRNA has the potential to target multiple mRNAs, it is generally involved in regulating various biological processes and signaling cascades.[42] In addition, the aberrant expression of miRNAs is reported in many diseases. Therefore, it is essential to identify key miRNAs and their target genes in the development of miRNA-based therapeutics in MB. Numerous databases such as DIANA-TarBase,[43] miRBase,[44] miRanda,[45] and HMDD v2.0[46] are available to predict miRNA-mRNA interaction, but the possibility of false prediction is high. Also, none of these sequence-based miRNA target prediction algorithms are designed for condition-specific or disease-specific prediction and, therefore, cannot avoid the problem of false-positive prediction. To overcome these limitations, a gene-expression-based miRNA target is needed for MB. Since single miRNA can regulate multiple gene expressions, it is potentially harmful to the patient to modulate multiple transcripts in miRNA-based therapeutics.[47] The miRNA targeting multiple genes contributes to both efficacy and side effects. However, the "many-to-many" relationship between miRNA and mRNA results in an extensive and robust regulatory network, which can have a powerful effect on cell proliferation and disease processes.[48]

It is difficult to determine the primary function of a given miRNA. Hence, the global transcriptomic data from the same experimental set are needed.[22] It is essential to establish the comprehensive downstream analysis of miRNA-mRNA interactions before applying the miRNA-based therapeutics. Since there are many factors that affect MB prognosis, the transcriptomic analysis is being evaluated experimentally to key tumor suppressor genes and oncogenes that are

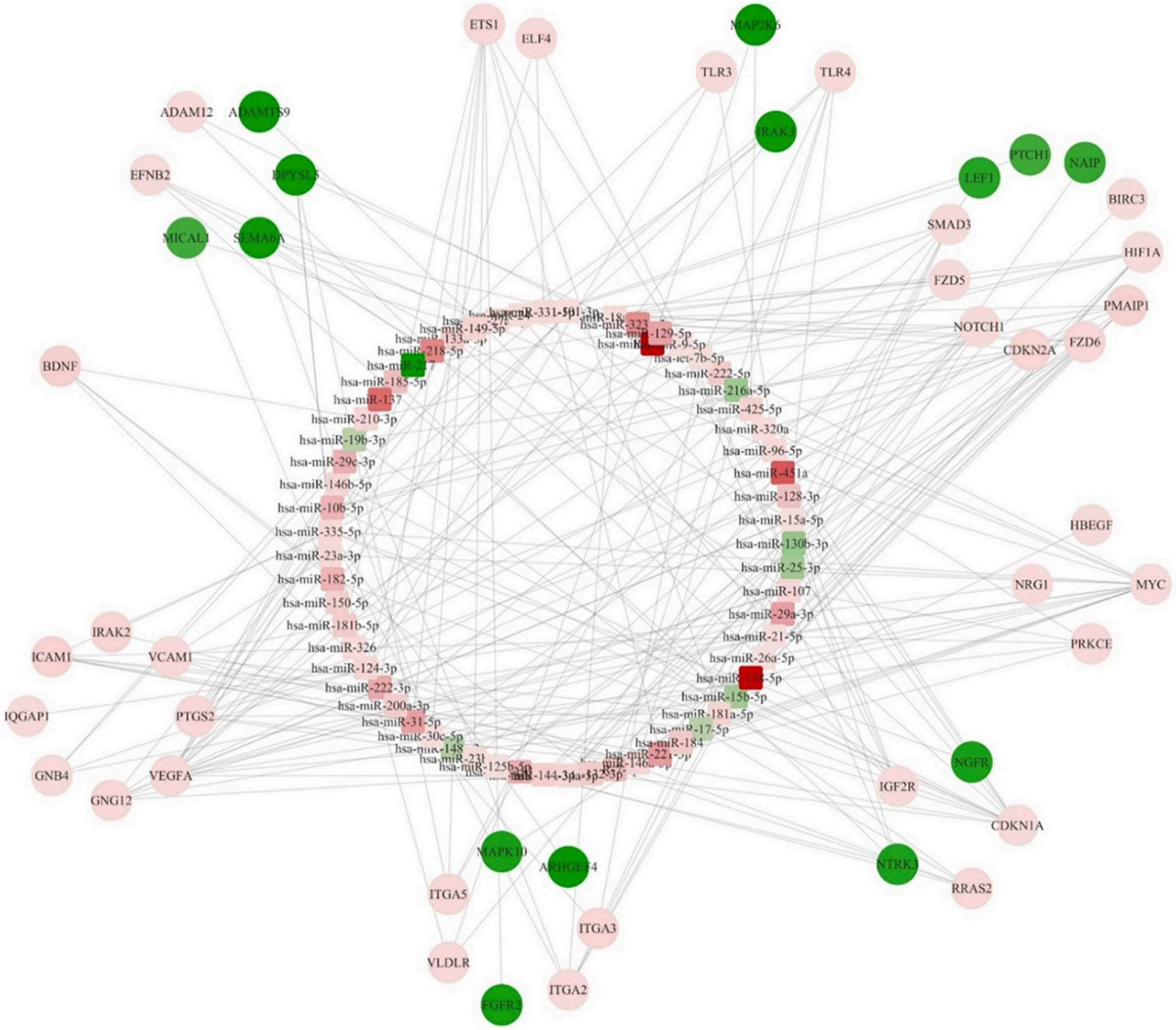

Figure 4. Molecular Network Identified by IPA between mRNA and miRNA in MB

The rectangle represents the miRNAs, and circles represent the mRNAs. Green represents upregulated miRNA and genes, and red represents downregulated miRNA and genes. The intensity of the respective colors indicates the fold expression (more intense green indicates more upregulation and vice versa; similar is for red annotation).

important for MB growth and progression.[49] RNA-seq has been used in transcriptome profiling of many cancers, including breast,[50] colon,[51] and pancreatic cancer.[52] Previous studies of large-scale gene expression analysis have used a microarray approach to identify tumor markers and potential therapeutic targets for MB.[53] However, microarray analyses have limited sensitivity.[54] RNA-seq is a comparatively newer approach to study the transcriptome profiling and provides a more accurate and sensitive measurement of transcript and transcript isoform levels. The RNA-seq technique is applied to many transcriptomes, including those in mouse and human cells.[55–61]

In MB, only a couple of studies have reported the IPA from transcriptomic data and miRNA profiling in tissue samples of the same patient. Pathway analysis suggests that miR-217 could be a potential oncogenic miRNA that negatively regulates the SIRT1, SMAD7, ROBO1, and FOXO3 genes, which are involved in tumor progression by diverse mechanisms (Figure 6). It has been reported that miR-217 was dysregulated in many tumor types.[30,62,63] However, the clinical significance and biological role of miR-217 in MB have not been explored. Our *in vitro* results shows that antimiR-217 reduces cell growth, migration, invasion, and induces apoptosis (Figure 7). These findings suggest that the

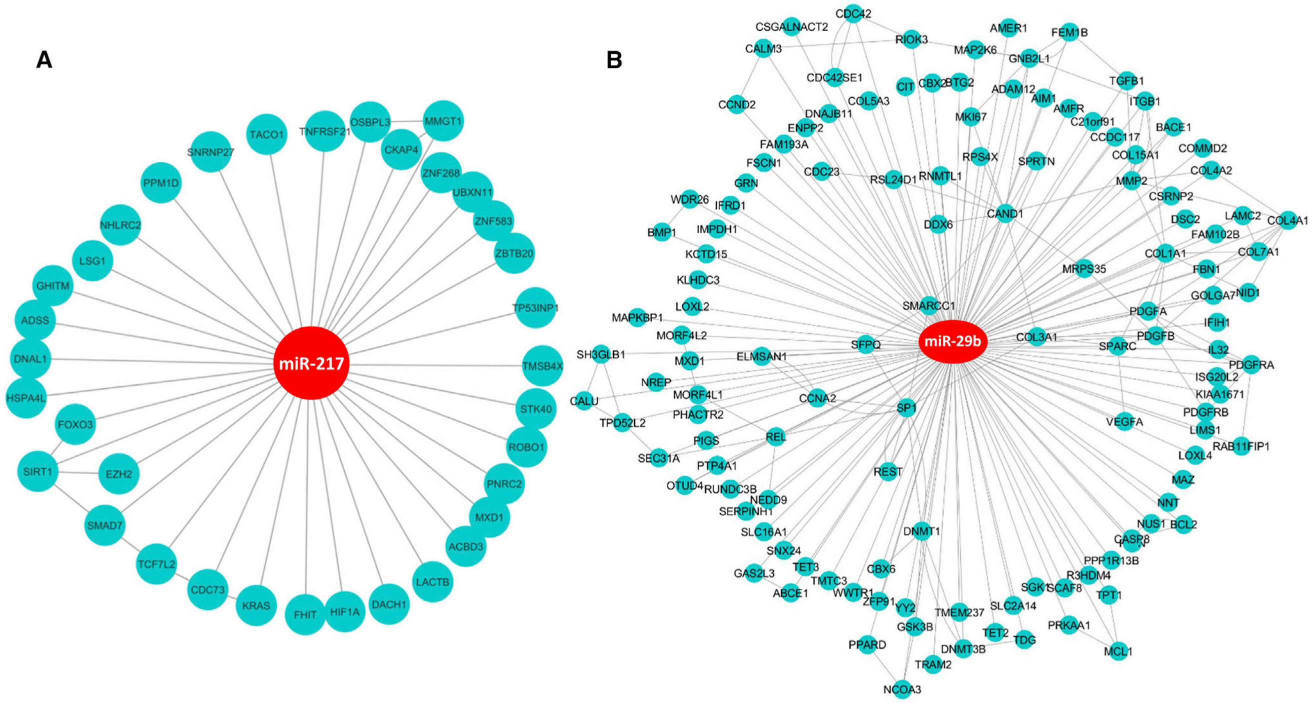

Figure 5. IPA of the Most Up- and Downregulated miRNAs and Their Target Genes
(A) miR-217. (B) miR-29b.

downregulation of miR-217 has significant therapeutic potential in group 3 MB. Also, IPA revealed several canonical pathways and molecular functions that are associated with MB (Figure 8A; Tables S4 and S5). Using the RNA-seq technique, we identified many more DEGs compared to normal cerebellar tissues (Figure 3). Our findings from the RNA-seq, RT-PCR, and IHC experiments provide additional support for the potential role of miRNAs in MB (Figure 7). This mRNA-miRNA correlation approach will help to eliminate false-positive targets of miRNA. Further, in future experiments, we will aim at identifying MB mRNA-miRNA interactions that are significantly associated with survival. The data from this study will allow us to minimize off-target effects and enhance therapeutic efficacy as new experimental therapies are developed (Figure 8B).

Our approach is distinct, and one of the very few to assess miRNA expression and then integrate these miRNA expression profiles with mRNA gene expression data from the same human primary MB specimens (Figure 8B). We identified many miRNA-mRNA regulatory networks in MB controlling various key pathways relevant to tumor development, progression, and therapy failure. We aimed to determine miRNA-regulated networks of gene expression in human primary MB specimens to gain insights of MB pathophysiology and potential pathways to modify the disease for therapeutic benefit. The profiling of miRNAs, short non-coding RNAs, and regulation of RNA target genes has been successful in other cancers but remains unclear in MB.

MATERIALS AND METHODS

Patient Cohorts

Surgical specimens of ten (five MB and five normal cerebellar) human tissue samples with clinical outcomes were obtained from the tissue bank facility of the University of Nebraska Medical Center (UNMC; Omaha, NE, USA). Information on patient's demographics, tumor location, histopathologic tumor type, and tumor stage were obtained from a pathology report provided by the tissue bank facility of the UNMC. The clinical and histopathological details of the patients are summarized in Table S5. The tissue localized adjacent to the tumor was considered as normal tissue. The tissue samples were stored at −80°C until used.

RNA Extraction

Total RNA from 10 frozen human patient tissues were extracted using QIAzol lysis reagent (QIAGEN, Germantown, MD, USA), according to the manufacturer's instructions. Briefly, 10–15 mg of tissues were dissolved in 700 μL QIAzol lysis reagent. It was then homogenized with ceramic beads in TissueRuptor for 10–20 s or until uniformly homogenized. Samples were then centrifuged for 5 min at 6,000 × g at room temperature. The supernatant was collected, and chloroform (140 μL) was added and vigorously mixed, followed by incubation at room temperature. The remaining procedures were followed with the RNeasy Mini Kit per the manufacturer's instructions (QIAGEN, Germantown, MD, USA). Total RNA concentration (260:280 nm ratio) was determined using BioTek's Microplate Spectrophotometer. RNA samples were stored at −80°C until further use.

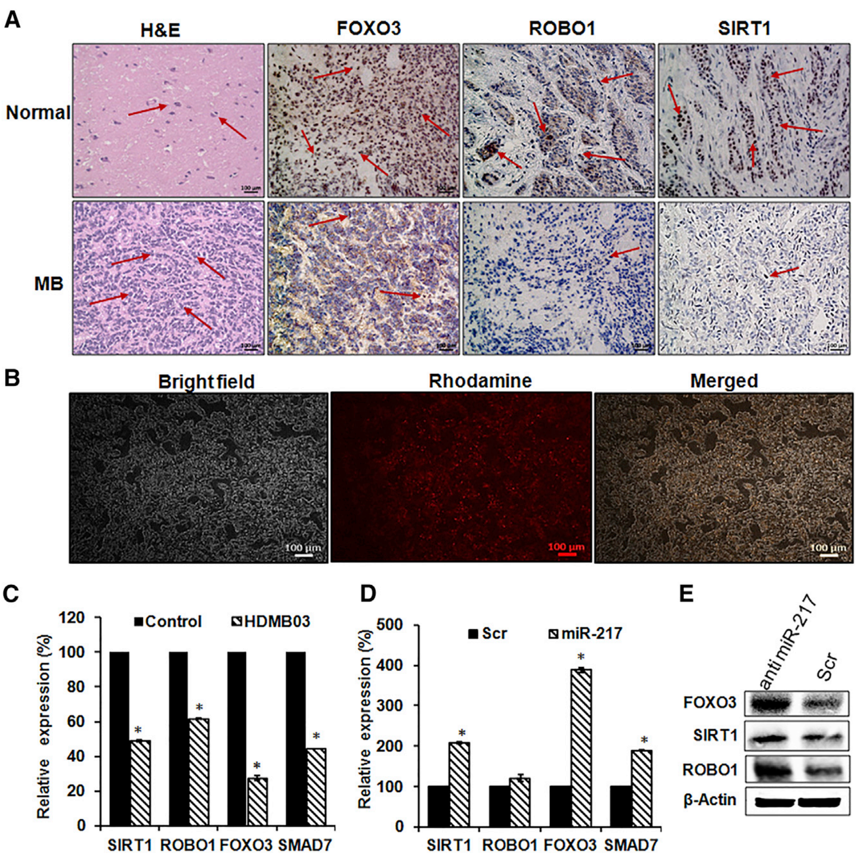

Figure 6. Expression and Validation of Various Targets of miR-217

(A) H&E and immunohistochemical staining for FOXO3, ROBO1, and SIRT1 protein in normal and MB patient tissues. Scale bars, 100 µm. Arrows indicate the stained cells. (B) Transfection of a labeled oligonucleotide using Lipofectamine RNAiMAX in HDMB03 cells. (C) The expression levels of FOXO3, ROBO1, SIRT1, and SMAD7 gene were measured and compared with that of the control cell line (SVG p12). Validation of regulation of miR-217 targets by (D) real-time PCR and (E) western blot in the HDMB03 cell line. The expression level of each gene and protein was measure and compared with scrambled (Scr) miR-217- and antimiR-217-transfected cells. The RT-PCR was performed with gene-specific primers. The expression of each gene was normalized with the average expression of the endogenous reference gene GAPDH. In western blot analysis, β-actin was used as a loading control. Data are shown as the mean ± SD of three separate experiments (Student's t test; $^*p < 0.05$; n = 3).

miRNA Expression Profiling

For miRNA profiling, cDNA was synthesized using the miScript II RT Kit (QIAGEN, MD) per the manufacturer's instructions. miRNA profiling was performed using the SYBR Green miScript miRNA PCR Array (catalog no. MIHS-108ZF; QIAGEN, Germantown, MD, USA), which could detect 84 previously identified miRNAs reported in various human brain cancers, of which 48 miRNAs were specific to human MB. The housekeeping genes (SNORD61, SNORD68, SNORD72, SNORD95, SNORD96A, and RNU6B/RNU6-2) were also included in the PCR array. The miScript miRNA PCR array plate was run on real-time PCR (Roche Light Cycler 480) to perform miRNA profiling. The expression of individual miRNAs was calculated by using crossing point (Cp) values. Unaffected normal cerebellar tissues were considered as controls.

RNA-Seq Sample Preparation and Sequencing

The quality, quantity, and integrity of the extracted total RNA were evaluated using an RNA pico chip, BioAnalyzer 2100 (Agilent Technologies, Santa Clara, CA, USA). Samples with an RNA quality score (RQN; an algorithm for judging the integrity of RNA samples) of >5.0 was used in RNA-seq (Figure S1). The starting amount was 1 µg total RNA. The mRNA was used to prepare cDNA libraries, using the TruSeq RNA Sample Preparation V2 Kit (Illumina, San Diego, CA, USA). The libraries were sequenced on an Illumina NextSeq 500 instrument with a 75-bp paired-end protocol at the sequencing core facility at the UNMC. A total of six libraries (three from MB and three from normal cerebellar tissues) from six patient samples were sequenced.

The quality of the sequencing data was analyzed by the bioinformatics team at the UNMC Bioinformatics and Systems Biology Core facility. The differential expression analysis between the MB and normal cerebellar samples was also performed. All the results were generated by utilizing STAR as the aligner and RSEM as the tool for annotation and quantification at both the gene and isoform levels.[33–35,64–66] The FPKM (fragments per kilobase of transcript per million mapped reads) value, a representation of the normalized expression value for each gene and/or isoform, was used as a measure of relative transcript abundance.

Biological Interpretation and Pathway Analysis

The differential expression of genes and its correlation with miRNAs were performed using IPA (Ingenuity Systems, Redwood City, CA, USA), a web-delivered application used to discover, visualize, and explore relevant networks. After performing statistical analysis with a $p < 0.05$, IPA was used to generate a heatmap for human genes. The multiple corrections were performed using the Benjamini-Hochberg procedure to identify statistically significant pathways and biological functions.[66] The significant genes were categorized, compared to functional categories in the IPA database, and ranked according to their p values. The p values less than 0.05 were considered statistically significant. The interaction between genes and miRNA was then queried within IPA to generate direct interaction networks that were overlapping.

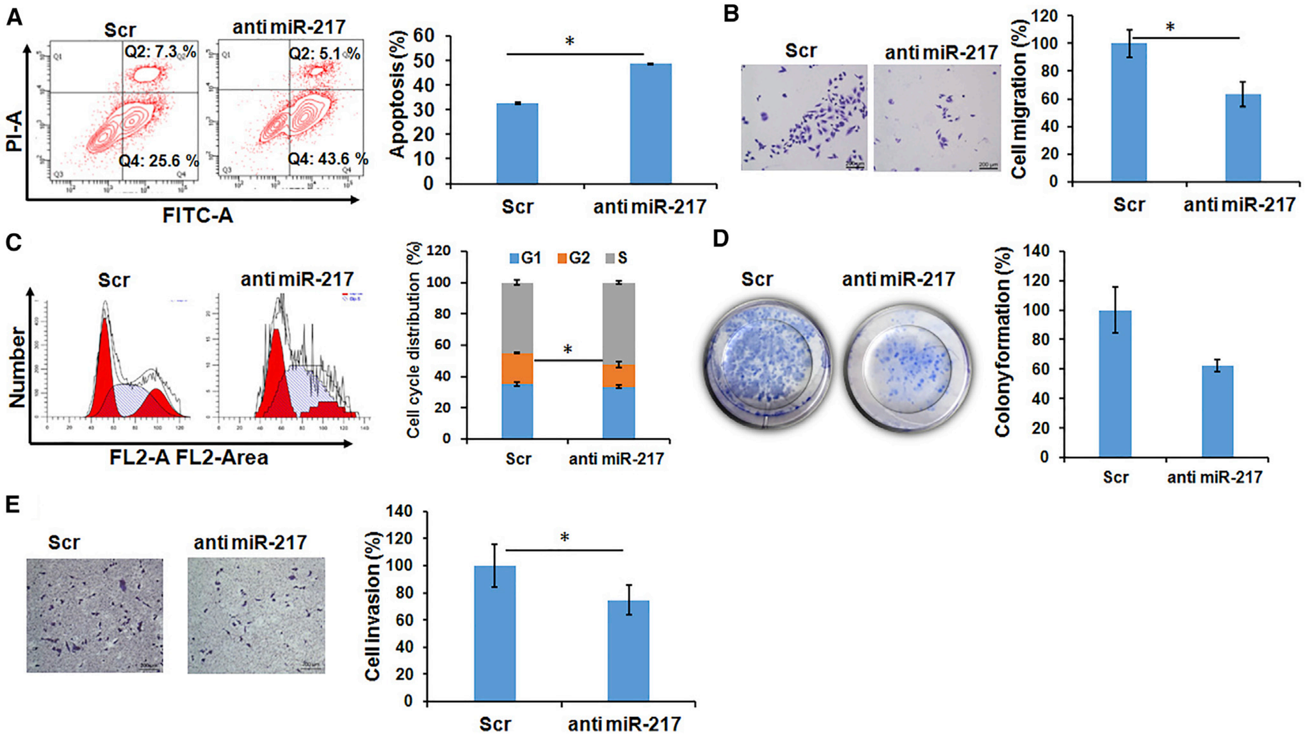

Figure 7. Tumorigenic Effect of miR-217

The inhibition of miR-217 (A) increases apoptosis, (B) decreases cell migration, (C) increases the percentage of the cell population in S phase, (D) decreases colony formation, and (E) decreases invasion in the HDMB03 cell line. Data are shown as the mean ± SD of three separate experiments (Student's t test; *$p < 0.05$; $n = 3$).

Cell Lines, Culture Condition, and Measurement of miR-217 Expression Level

Daoy and SVG p12 cell lines were purchased from the ATCC and cultured in Eagle's minimum essential medium (EMEM) containing 10% fetal bovine serum (FBS) and 1% penicillin-streptomycin in a humidified 37°C incubator supplemented with 5% CO_2. The HDMB03 cell line was provided by Dr. Nagendra K. Chaturvedi, while NPCs and the D341 cell line were provided by Dr. Sidharth Mahapatra from the University of Nebraska Medical Center, Omaha, NE, USA. HDMB03 and D341 cell lines were cultured in DMEM containing 10% and 20% FBS, respectively, with 1% penicillin-streptomycin. The NPC cell line was cultured in neural progenitor medium 2 (Stem Cell Technologies).

Total RNA from Daoy, SVG p12, NPC, HDMB03, and D341 cell lines were extracted using an RNA isolation kit (QIAGEN, Germantown, MD, USA) followed by reverse transcription into a cDNA template.[67–69] For quantification of miR-217 in these cells, the miScript primer assays were used per the manufacturer's instructions (QIAGEN, Germantown, MD, USA). U6 was used as the internal control.

Real-Time qPCR, Transfection, Immunohistochemical Staining, and Western Blot Analysis

The mRNA expression levels were validated in the HDMB03 cell line using the SYBR Green PCR Master Mix Reagent (Applied Biosystems) and LightCycler 480 machine (Roche). The levels of mRNA were determined from cDNA by using the following primers: SIRT1 (forward; F) 5′-GCTGGCCTAATAGAGTGGCAA-3′ and (reverse; R) 5′-CTCAGCGCCATGGAAAATG-3′; SMAD7 (F) 5′-GAATCTTACGGGAAGATCAACCC-3′ and (R) 5′-CGCAGAGTCGGCTAAGGTG-3′; FOXO3 (F) 5′-ACGGTGTTCGGACCTTCATC-3′ and (R) 5′-TGCTGGCCTGAGACATCAAG-3′; ROBO1 (F) 5′-GTTGTGGCCAATGTCGAAAC-3′ and (R) 5′-GTTTTGGTGAACACCAGCCT-3′; and GAPDH (F) 5′-ACCACAGTCCATGCCATCAC-3′ and (R) 5′-TCCACCACCCTGTTGCTGTA-3′.

The scrambled miRNA (negative control) and antimiR-217 were purchased from Ambion (Life Technologies). Cells were transfected with antimiR-217 and scrambled miRNA using Lipofectamine RNAiMAX reagent (Invitrogen) as described earlier.[68–70] Labeled oligonucleotide coupled with Lipofectamine RNAiMAX was also used as positive control to ensure the efficiency of transfection.

Human MB patient and normal tissues were stained for FOXO3 (LifeSpan BioSciences), ROBO1 (Santa Cruz Biotechnology), and SIRT1 (Santa Cruz) antibody (1:50 dilution) to determine the expression levels. Positively stained color (brown) was selected for comparison of relative intensity.

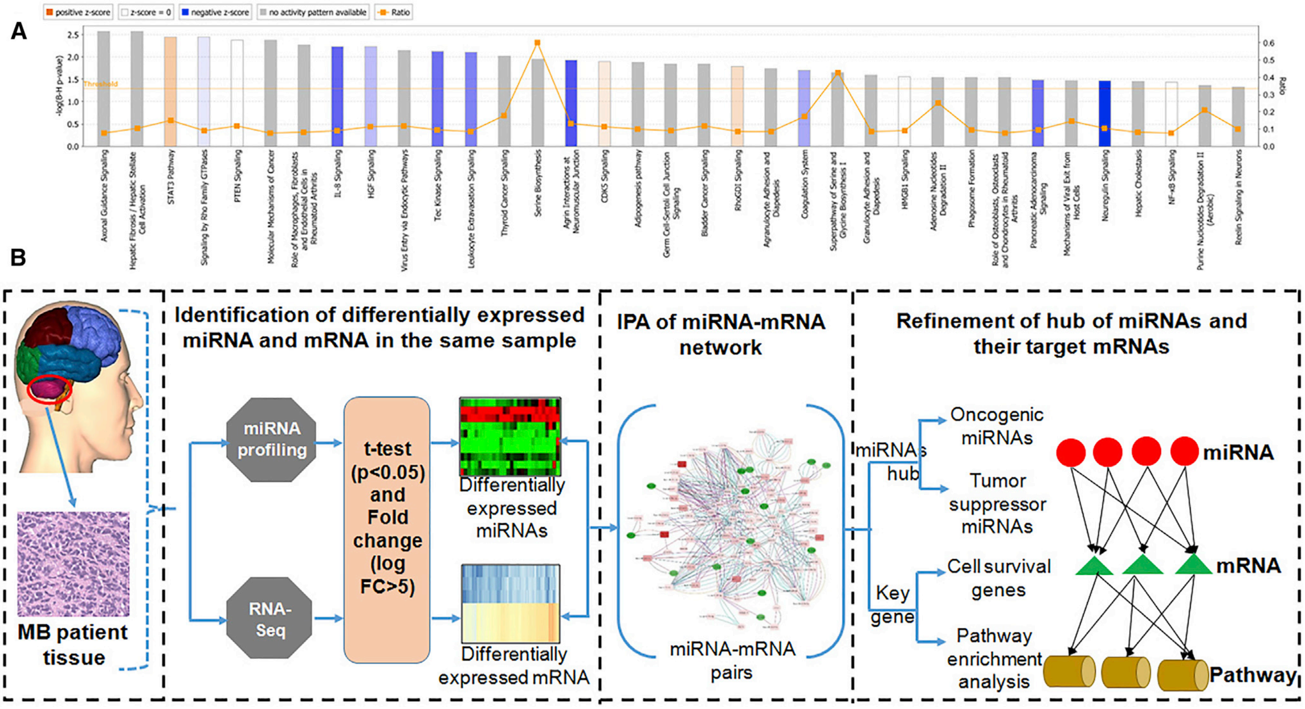

Figure 8. Functional Analysis of the miRNA-mRNA Network

(A) IPA of differentially expressed pathways in an MB specimen specific to the cerebellum. Higher (−)log Benjamini-Hochberg (B-H) p value corresponds to the more significant pathway. The solid yellow line in the bar charts reflects the ratio that refers to the number of molecules from the dataset that map to the pathway divided by the total number of molecules that map the canonical pathway from the IPA knowledge base. *Z* score is the activation score for a pathway or disease or function. The *Z* score reflects how much the pathway or function is activated (orange) or deactivated (blue) due to the changes in the expression of genes involved in those pathways or functions. (B) Illustration of the design of miRNA-mRNA target binding in a human MB specimen. Expression profiles of miRNAs and mRNA and their correlation in the same MB sample are used to discover the complex miRNA-mRNA interaction.

For western blot analysis, the HDMB03 cells transfected with scrambled and antimiR-217 were lysed with radioimmunoprecipitation assay (RIPA) buffer. The same concentration of scrambled and antimiR-217 protein was transferred to the polyvinylidene fluoride (PVDF) membrane by the iBlot gel transfer system (Life Technologies, Carlsbad, CA, USA). The membrane was incubated with primary and secondary antibodies. Target proteins were detected by the ImmunoCruz Western Blotting Luminol Reagent Kit (Santa Cruz Biotechnology).[69] The primary antibodies (1:1,000 dilution) used were the following: anti-Robo1 (Santa Cruz, sc-293444), anti-FOXO3 (LifeSpan Biosciences, LS-B1814), anti-SIRT1 (Santa Cruz, sc15404), and anti-β-actin (Santa Cruz, sc-47778).

Apoptosis and Cell-Cycle Analysis

3×10^5 HDMB03 cells were transfected with scrambled and antimiR-217 and were grown in a 6-well plate for 72 hr. After 72 hr, both live and dead cells were collected and washed with cold PBS. For apoptosis assay, the cell pellets were suspended in 500 μL binding buffer containing 5 μL annexin-V fluorescein isothiocyanate (FITC) and 1 μL propidium iodide (Alexa Fluor 488 Annexin V/Dead Cell Apoptosis Kit, Invitrogen). The mixture was incubated for 15 min in the dark and analyzed by flow cytometry. For cell-cycle analysis, the HDMB03 cell pellets were suspended in 70% ethanol and incubated overnight. The cells were washed with PBS and suspended in FxCycle PI/RNase staining solution (Molecular Probes) and analyzed with a FACS Calibur flow cytometer after incubation at 37°C for 30 min in the dark.

Cell Migration, Invasion, and Clonogenic Assay

The effect of antimiR-217 on the cell migration and invasion was done by using the Transwell membrane filter insert in a 6-well culture plate. For migration assay, HDMB03 cells transfected with scrambled and antimiR-217 were seeded into the upper chambers of a Transwell insert in DMEM supplemented with 1% FBS. The lower chambers were filled with DMEM supplemented with 10% FBS. Cells were cultured for 72 hr. The number of migrated cells was quantified after crystal violet staining for 10 min. For the invasion assay, 200 μL Matrigel (BD Biosciences, San Jose, CA, USA) was added to each Transwell insert with DMEM containing 1% FBS. The number of cells that invaded Matrigel was quantified after staining with crystal violet. For the clonogenic assay, HDMB03 cells transfected with scrambled miRNA and antimiR-217 were seeded into a 6-well plate. Each well had 500 cells. After 12 days of post-incubation, colonies in each well were fixed in 10% formalin, stained with crystal violet, quantified, and visualized.

Statistical Analysis

Student's unpaired t test was used to compare the mean values of individual groups. A p value <0.05 was considered as statistically significant.

SUPPLEMENTAL INFORMATION

Supplemental Information includes three figures and six tables and can be found with this article online at https://doi.org/10.1016/j.omtn.2018.06.004.

AUTHOR CONTRIBUTIONS

Vinod Kumar, Virender Kumar, A.K.C., D.W.C., T.M., and R.I.M. conceived and designed the study. Vinod Kumar, Virender Kumar, and A.K.C. conducted the experiments. Vinod Kumar, Virender Kumar, A.K.C., T.M., and R.I.M. analyzed the data and prepared the manuscript. All authors read and approved the manuscript.

CONFLICTS OF INTERESTS

The authors declare that they have no competing interests.

ACKNOWLEDGMENTS

We greatly acknowledge the financial support of the Pediatric Cancer Research Group of the University of Nebraska Medical Center and Children's Hospital (LB805) and The Team Jack Foundation for this work. We would also like to thank the Bioinformatics and Systems Biology Core at UNMC for providing RNA-seq data analysis services, which receives support from the Nebraska Research Initiative (NRI) and NIH (2P20GM103427 and 5P30CA036727).

REFERENCES

1. Louis, D.N., Ohgaki, H., Wiestler, O.D., Cavenee, W.K., Burger, P.C., Jouvet, A., Scheithauer, B.W., and Kleihues, P. (2007). The 2007 WHO classification of tumours of the central nervous system. Acta Neuropathol. *114*, 97–109.
2. Schüller, U., Koch, A., Hartmann, W., Garrè, M.L., Goodyer, C.G., Cama, A., Sörensen, N., Wiestler, O.D., and Pietsch, T. (2005). Subtype-specific expression and genetic alterations of the chemokinereceptor gene CXCR4 in medulloblastomas. Int. J. Cancer *117*, 82–89.
3. Northcott, P.A., Korshunov, A., Witt, H., Hielscher, T., Eberhart, C.G., Mack, S., Bouffet, E., Clifford, S.C., Hawkins, C.E., French, P., et al. (2011). Medulloblastoma comprises four distinct molecular variants. J. Clin. Oncol. *29*, 1408–1414.
4. Jones, D.T., Jäger, N., Kool, M., Zichner, T., Hutter, B., Sultan, M., Cho, Y.J., Pugh, T.J., Hovestadt, V., Stütz, A.M., et al. (2012). Dissecting the genomic complexity underlying medulloblastoma. Nature *488*, 100–105.
5. Northcott, P.A., Jones, D.T., Kool, M., Robinson, G.W., Gilbertson, R.J., Cho, Y.J., Pomeroy, S.L., Korshunov, A., Lichter, P., Taylor, M.D., and Pfister, S.M. (2012). Medulloblastomics: the end of the beginning. Nat. Rev. Cancer *12*, 818–834.
6. Schwalbe, E.C., Lindsey, J.C., Nakjang, S., Crosier, S., Smith, A.J., Hicks, D., Rafiee, G., Hill, R.M., Iliasova, A., Stone, T., et al. (2017). Novel molecular subgroups for clinical classification and outcome prediction in childhood medulloblastoma: a cohort study. Lancet Oncol. *18*, 958–971.
7. Cavalli, F.M.G., Remke, M., Rampasek, L., Peacock, J., Shih, D.J.H., Luu, B., Garzia, L., Torchia, J., Nor, C., Morrissy, A.S., et al. (2017). Intertumoral heterogeneity within medulloblastoma subgroups. Cancer Cell *31*, 737–754.e6.
8. Lin, C.Y., Erkek, S., Tong, Y., Yin, L., Federation, A.J., Zapatka, M., Haldipur, P., Kawauchi, D., Risch, T., Warnatz, H.J., et al. (2016). Active medulloblastoma enhancers reveal subgroup-specific cellular origins. Nature *530*, 57–62.
9. Gibson, P., Tong, Y., Robinson, G., Thompson, M.C., Currle, D.S., Eden, C., Kranenburg, T.A., Hogg, T., Poppleton, H., Martin, J., et al. (2010). Subtypes of medulloblastoma have distinct developmental origins. Nature *468*, 1095–1099.
10. Kumar, V., Kumar, V., McGuire, T., Coulter, D.W., Sharp, J.G., and Mahato, R.I. (2017). Challenges and recent advances in medulloblastoma therapy. Trends Pharmacol. Sci. *38*, 1061–1084.
11. Taylor, M.D., Northcott, P.A., Korshunov, A., Remke, M., Cho, Y.J., Clifford, S.C., Eberhart, C.G., Parsons, D.W., Rutkowski, S., Gajjar, A., et al. (2012). Molecular subgroups of medulloblastoma: the current consensus. Acta Neuropathol. *123*, 465–472.
12. Gilbertson, R.J., and Ellison, D.W. (2008). The origins of medulloblastoma subtypes. Annu. Rev. Pathol. *3*, 341–365.
13. Bartel, D.P. (2004). MicroRNAs: genomics, biogenesis, mechanism, and function. Cell *116*, 281–297.
14. Kumar, V., Mondal, G., Slavik, P., Rachagani, S., Batra, S.K., and Mahato, R.I. (2015). Codelivery of small molecule hedgehog inhibitor and miRNA for treating pancreatic cancer. Mol. Pharm. *12*, 1289–1298.
15. Murchison, E.P., and Hannon, G.J. (2004). miRNAs on the move: miRNA biogenesis and the RNAi machinery. Curr. Opin. Cell Biol. *16*, 223–229.
16. Eulalio, A., Huntzinger, E., and Izaurralde, E. (2008). Getting to the root of miRNA-mediated gene silencing. Cell *132*, 9–14.
17. Filipowicz, W., Bhattacharyya, S.N., and Sonenberg, N. (2008). Mechanisms of post-transcriptional regulation by microRNAs: are the answers in sight? Nat. Rev. Genet. *9*, 102–114.
18. Chekulaeva, M., and Filipowicz, W. (2009). Mechanisms of miRNA-mediated post-transcriptional regulation in animal cells. Curr. Opin. Cell Biol. *21*, 452–460.
19. Wilczynska, A., and Bushell, M. (2015). The complexity of miRNA-mediated repression. Cell Death Differ. *22*, 22–33.
20. Kumar, V., and Mahato, R.I. (2015). Delivery and targeting of miRNAs for treating liver fibrosis. Pharm. Res. *32*, 341–361.
21. Min, H., and Yoon, S. (2010). Got target? Computational methods for microRNA target prediction and their extension. Exp. Mol. Med. *42*, 233–244.
22. Wang, Z., Gerstein, M., and Snyder, M. (2009). RNA-seq: a revolutionary tool for transcriptomics. Nat. Rev. Genet. *10*, 57–63.
23. Marguerat, S., and Bähler, J. (2010). RNA-seq: from technology to biology. Cell. Mol. Life Sci. *67*, 569–579.
24. Mardis, E.R. (2008). The impact of next-generation sequencing technology on genetics. Trends Genet. *24*, 133–141.
25. Genovesi, L.A., Anderson, D., Carter, K.W., Giles, K.M., and Dallas, P.B. (2012). Identification of suitable endogenous control genes for microRNA expression profiling of childhood medulloblastoma and human neural stem cells. BMC Res. Notes *5*, 507.
26. Higdon, R., Kala, J., Wilkins, D., Yan, J.F., Sethi, M.K., Lin, L., Liu, S., Montague, E., Janko, I., Choiniere, J., et al. (2017). Integrated proteomic and transcriptomic-based approaches to identifying signature biomarkers and pathways for elucidation of Daoy and UW228 subtypes. Proteomes *5*, E5.
27. Genovesi, L.A., Carter, K.W., Gottardo, N.G., Giles, K.M., and Dallas, P.B. (2011). Integrated analysis of miRNA and mRNA expression in childhood medulloblastoma compared with neural stem cells. PLoS ONE *6*, e23935.
28. Bai, M., Zhang, M., Long, F., Yu, N., Zeng, A., and Wang, X. (2017). MiR-217 promotes cutaneous squamous cell carcinoma progression by targeting PTRF. Am. J. Transl. Res. *9*, 647–655.
29. Li, J., Li, D., and Zhang, W. (2016). Tumor suppressor role of miR-217 in human epithelial ovarian cancer by targeting IGF1R. Oncol. Rep. *35*, 1671–1679.
30. Zhang, Q., Yuan, Y., Cui, J., Xiao, T., and Jiang, D. (2015). MiR-217 promotes tumor proliferation in breast cancer via targeting DACH1. J. Cancer *6*, 184–191.
31. Zhang, J., Wang, C., and Xu, H. (2017). miR-217 suppresses proliferation and promotes apoptosis in cardiac myxoma by targeting interleukin-6. Biochem. Biophys. Res. Commun. *490*, 713–718.
32. Zhou, W., Song, F., Wu, Q., Liu, R., Wang, L., Liu, C., Peng, Y., Mao, S., Feng, J., and Chen, C. (2017). miR-217 inhibits triple-negative breast cancer cell growth, migration, and invasion through targeting KLF5. PLoS ONE *12*, e0176395.

33. Kanitz, A., Gypas, F., Gruber, A.J., Gruber, A.R., Martin, G., and Zavolan, M. (2015). Comparative assessment of methods for the computational inference of transcript isoform abundance from RNA-seq data. Genome Biol. *16*, 150.

34. Dobin, A., and Gingeras, T.R. (2015). Mapping RNA-seq reads with STAR. Curr. Protoc. Bioinformatics *51*, 11.14.1–11.14.19.

35. Soneson, C., Love, M.I., and Robinson, M.D. (2015). Differential analyses for RNA-seq: transcript-level estimates improve gene-level inferences. F1000Res. *4*, 1521.

36. Zhang, S., Liu, X., Liu, J., Guo, H., Xu, H., and Zhang, G. (2017). PGC-1 alpha interacts with microRNA-217 to functionally regulate breast cancer cell proliferation. Biomed. Pharmacother. *85*, 541–548.

37. Zhang, Q., Yuan, Y., Cui, J., Xiao, T., and Jiang, D. (2015). MiR-217 promotes tumor proliferation in breast cancer via targeting DACH1. J. Cancer *6*, 184–191.

38. Gajjar, A., Chintagumpala, M., Ashley, D., Kellie, S., Kun, L.E., Merchant, T.E., Woo, S., Wheeler, G., Ahern, V., Krasin, M.J., et al. (2006). Risk-adapted craniospinal radiotherapy followed by high-dose chemotherapy and stem-cell rescue in children with newly diagnosed medulloblastoma (St Jude Medulloblastoma-96): long-term results from a prospective, multicentre trial. Lancet Oncol. *7*, 813–820.

39. Gudrunardottir, T., Lannering, B., Remke, M., Taylor, M.D., Wells, E.M., Keating, R.F., and Packer, R.J. (2014). Treatment developments and the unfolding of the quality of life discussion in childhood medulloblastoma: a review. Childs Nerv. Syst. *30*, 979–990.

40. Guo, H., Ingolia, N.T., Weissman, J.S., and Bartel, D.P. (2010). Mammalian microRNAs predominantly act to decrease target mRNA levels. Nature *466*, 835–840.

41. Ferretti, E., De Smaele, E., Po, A., Di Marcotullio, L., Tosi, E., Espinola, M.S., Di Rocco, C., Riccardi, R., Giangaspero, F., Farcomeni, A., et al. (2009). MicroRNA profiling in human medulloblastoma. Int. J. Cancer *124*, 568–577.

42. Xue, J., Xie, F., Xu, J., Liu, Y., Liang, Y., Wen, Z., and Li, M. (2017). A new network-based strategy for predicting the potential miRNA-mRNA interactions in tumorigenesis. Int. J. Genomics *2017*, 3538568.

43. Vlachos, I.S., Paraskevopoulou, M.D., Karagkouni, D., Georgakilas, G., Vergoulis, T., Kanellos, I., Anastasopoulos, I.L., Maniou, S., Karathanou, K., Kalfakakou, D., et al. (2015). DIANA-TarBase v7.0: indexing more than half a million experimentally supported miRNA:mRNA interactions. Nucleic Acids Res. *43*, D153–D159.

44. Kozomara, A., and Griffiths-Jones, S. (2014). miRBase: annotating high confidence microRNAs using deep sequencing data. Nucleic Acids Res. *42*, D68–D73.

45. John, B., Enright, A.J., Aravin, A., Tuschl, T., Sander, C., and Marks, D.S. (2004). Human microRNA targets. PLoS Biol. *2*, e363.

46. Li, Y., Qiu, C., Tu, J., Geng, B., Yang, J., Jiang, T., and Cui, Q. (2014). HMDD v2.0: a database for experimentally supported human microRNA and disease associations. Nucleic Acids Res. *42*, D1070–D1074.

47. Hydbring, P., and Badalian-Very, G. (2013). Clinical applications of microRNAs [version 3]. F1000Res. *2*, 136.

48. Griffiths-Jones, S., Saini, H.K., van Dongen, S., and Enright, A.J. (2008). miRBase: tools for microRNA genomics. Nucleic Acids Res. *36*, D154–D158.

49. Hovestadt, V., Jones, D.T., Picelli, S., Wang, W., Kool, M., Northcott, P.A., Sultan, M., Stachurski, K., Ryzhova, M., Warnatz, H.J., et al. (2014). Decoding the regulatory landscape of medulloblastoma using DNA methylation sequencing. Nature *510*, 537–541.

50. Stephens, P.J., Tarpey, P.S., Davies, H., Van Loo, P., Greenman, C., Wedge, D.C., Nik-Zainal, S., Martin, S., Varela, I., Bignell, G.R., et al.; Oslo Breast Cancer Consortium (OSBREAC) (2012). The landscape of cancer genes and mutational processes in breast cancer. Nature *486*, 400–404.

51. Wu, Y., Wang, X., Wu, F., Huang, R., Xue, F., Liang, G., Tao, M., Cai, P., and Huang, Y. (2012). Transcriptome profiling of the cancer, adjacent non-tumor and distant normal tissues from a colorectal cancer patient by deep sequencing. PLoS ONE *7*, e41001.

52. Mao, Y., Shen, J., Lu, Y., Lin, K., Wang, H., Li, Y., Chang, P., Walker, M.G., and Li, D. (2017). RNA sequencing analyses reveal novel differentially expressed genes and pathways in pancreatic cancer. Oncotarget *8*, 42537–42547.

53. Ma, H.I., Kao, C.L., Lee, Y.Y., Chiou, G.Y., Tai, L.K., Lu, K.H., Huang, C.S., Chen, Y.W., Chiou, S.H., Cheng, I.C., and Wong, T.T. (2010). Differential expression profiling between atypical teratoid/rhabdoid and medulloblastoma tumor in vitro and in vivo using microarray analysis. Childs Nerv. Syst. *26*, 293–303.

54. Zhao, S., Fung-Leung, W.P., Bittner, A., Ngo, K., and Liu, X. (2014). Comparison of RNA-Seq and microarray in transcriptome profiling of activated T cells. PLoS ONE *9*, e78644.

55. Nagalakshmi, U., Wang, Z., Waern, K., Shou, C., Raha, D., Gerstein, M., and Snyder, M. (2008). The transcriptional landscape of the yeast genome defined by RNA sequencing. Science *320*, 1344–1349.

56. Wilhelm, B.T., Marguerat, S., Watt, S., Schubert, F., Wood, V., Goodhead, I., Penkett, C.J., Rogers, J., and Bähler, J. (2008). Dynamic repertoire of a eukaryotic transcriptome surveyed at single-nucleotide resolution. Nature *453*, 1239–1243.

57. Mortazavi, A., Williams, B.A., McCue, K., Schaeffer, L., and Wold, B. (2008). Mapping and quantifying mammalian transcriptomes by RNA-seq. Nat. Methods *5*, 621–628.

58. Lister, R., O'Malley, R.C., Tonti-Filippini, J., Gregory, B.D., Berry, C.C., Millar, A.H., and Ecker, J.R. (2008). Highly integrated single-base resolution maps of the epigenome in *Arabidopsis*. Cell *133*, 523–536.

59. Cloonan, N., Forrest, A.R., Kolle, G., Gardiner, B.B., Faulkner, G.J., Brown, M.K., Taylor, D.F., Steptoe, A.L., Wani, S., Bethel, G., et al. (2008). Stem cell transcriptome profiling via massive-scale mRNA sequencing. Nat. Methods *5*, 613–619.

60. Marioni, J.C., Mason, C.E., Mane, S.M., Stephens, M., and Gilad, Y. (2008). RNA-seq: an assessment of technical reproducibility and comparison with gene expression arrays. Genome Res. *18*, 1509–1517.

61. Morin, R., Bainbridge, M., Fejes, A., Hirst, M., Krzywinski, M., Pugh, T., McDonald, H., Varhol, R., Jones, S., and Marra, M. (2008). Profiling the HeLa S3 transcriptome using randomly primed cDNA and massively parallel short-read sequencing. Biotechniques *45*, 81–94.

62. Chen, D.L., Zhang, D.S., Lu, Y.X., Chen, L.Z., Zeng, Z.L., He, M.M., Wang, F.H., Li, Y.H., Zhang, H.Z., Pelicano, H., et al. (2015). microRNA-217 inhibits tumor progression and metastasis by downregulating EZH2 and predicts favorable prognosis in gastric cancer. Oncotarget *6*, 10868–10879.

63. Li, H., Zhao, J., Zhang, J.W., Huang, Q.Y., Huang, J.Z., Chi, L.S., Tang, H.J., Liu, G.Q., Zhu, D.J., and Ma, W.M. (2013). MicroRNA-217, down-regulated in clear cell renal cell carcinoma and associated with lower survival, suppresses cell proliferation and migration. Neoplasma *60*, 511–515.

64. Teng, M., Love, M.I., Davis, C.A., Djebali, S., Dobin, A., Graveley, B.R., Li, S., Mason, C.E., Olson, S., Pervouchine, D., et al. (2016). A benchmark for RNA-seq quantification pipelines. Genome Biol. *17*, 74.

65. Zhang, C., Zhang, B., Lin, L.L., and Zhao, S. (2017). Evaluation and comparison of computational tools for RNA-seq isoform quantification. BMC Genomics *18*, 583.

66. Noble, W.S. (2009). How does multiple testing correction work? Nat. Biotechnol. *27*, 1135–1137.

67. Wen, D., Peng, Y., Lin, F., Singh, R.K., and Mahato, R.I. (2017). Micellar delivery of miR-34a modulator rubone and paclitaxel in resistant prostate cancer. Cancer Res. *77*, 3244–3254.

68. Singh, S., Chitkara, D., Kumar, V., Behrman, S.W., and Mahato, R.I. (2013). miRNA profiling in pancreatic cancer and restoration of chemosensitivity. Cancer Lett. *334*, 211–220.

69. Kumar, V., Chaudhary, A.K., Dong, Y., Zhong, H.A., Mondal, G., Lin, F., et al. (2017). Design, synthesis and biological evaluation of novel Hedgehog inhibitors for treating pancreatic cancer. Sci. Rep. *7*, 1665.

70. Chaudhary, A.K., Mondal, G., Kumar, V., Kattel, K., and Mahato, R.I. (2017). Chemosensitization and inhibition of pancreatic cancer stem cell proliferation by overexpression of microRNA-205. Cancer Lett. *402*, 1–8.

OMTN, Volume *12*

Supplemental Information

Impact of miRNA-mRNA Profiling and Their Correlation on Medulloblastoma Tumorigenesis

Vinod Kumar, Virender Kumar, Amit Kumar Chaudhary, Donald W. Coulter, Timothy McGuire, and Ram I. Mahato

Supplemental information

Supplemental figures

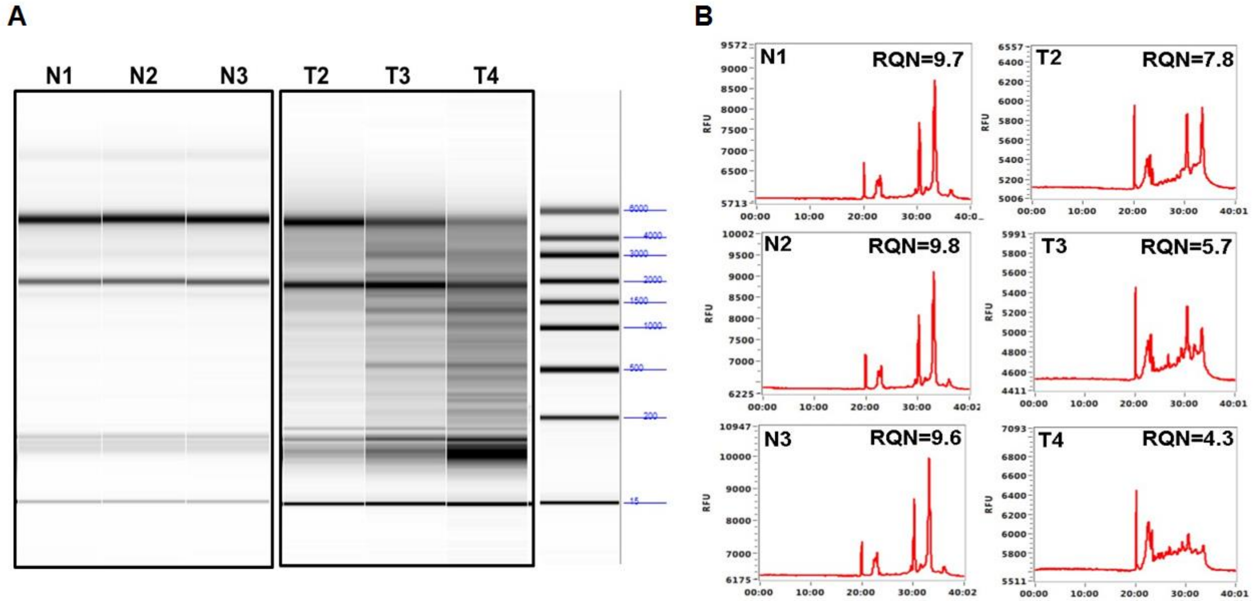

Figure S1. Electropherogram detailing of the RNA sample. (**A**) Gel image of total RNA samples, analyzed on the Agilent 2100 Bioanalyzer System using the Eukaryote Total RNA Nano assay. (**B**) The RNA quality score (RQN) was used to test the intactness and quality of RNA samples used in the RNA-Seq analysis.

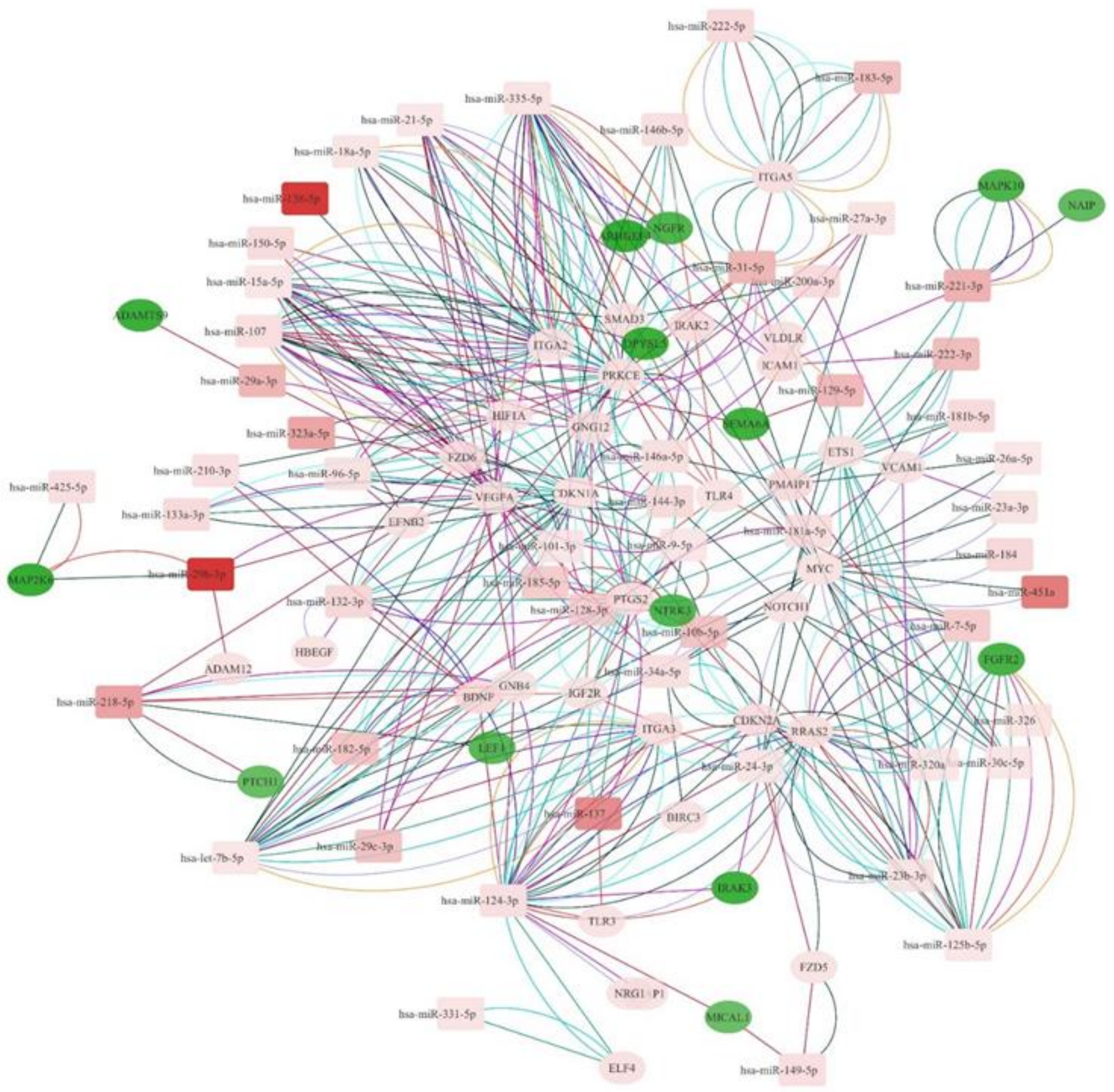

Figure S2. Molecular network identified by IPA between miRNA and mRNA in MB.

The rectangle represents the miRNAs and circles mRNAs.

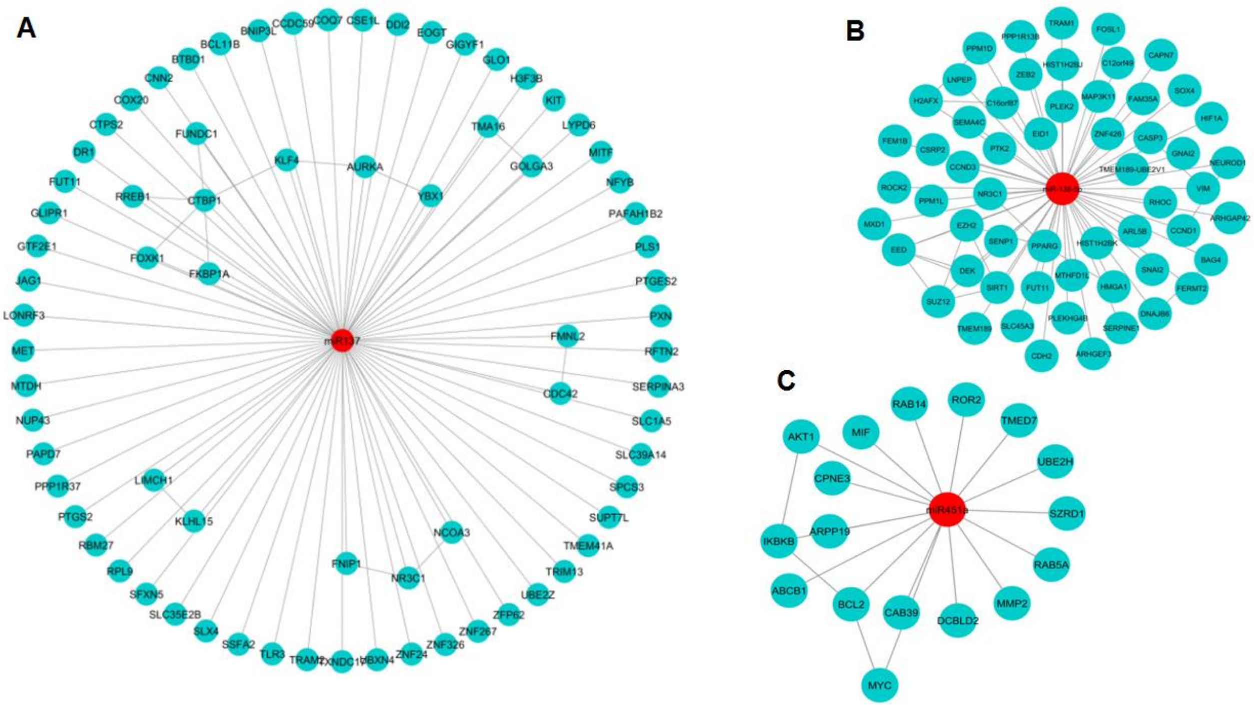

Figure S3. IPA of the most deregulated miRNAs and their target genes. (A). miR-137. **(B).** miR-138-5p. **(C).** miR-451a.

Description of Supplementary Tables

Supplementary Table 1S. Comparison of gene expression profile in medulloblastoma and normal cerebellar tissues obtain from RNA-Seq.

Supplementary Table 2S. The number of genes and canonical pathways specific to the cerebellum. The log fold change five or above was considered as significant.

Supplementary Table 3S. The number of genes involved in various diseases and pathways specific to the cerebellum.

Supplementary Table 4S. Top diseases and functions controlled by the genes which express in the cerebellum. The log fold change five or above was considered as significant.

Supplementary Table 5S.

(A) Clinical data and molecular sub-type of primary MB specimens utilized in this investigation

(B) The top five cellular function and top upstream regulators and their predicted activation from IPA in MB specimens

Supplementary Table 6S. The mRNA and miRNA target correlation. The red color depicts the positive correlation and green color negative correlation.

Supplementary Table 5 S.

(A) Clinical data and molecular sub-type of primary MB specimens utilized in this investigation.

**Variables**	
Sample size	10
Age (years)	Median (20.8)
	Range (8-46)
Gender	Male (4)
	Female (6)
Desmoplasia	2
Tumor grade	WHO grade IV (4)
	WHO grade II (1)
Metastasis state	No

(B) The top five cellular function and top upstream regulators and their predicted activation from IPA in MB specimens.

**Top molecular and cellular functions**		
**Molecular and cellular functions**	**Molecules**	**p-value**
Cellular movement	157	1.83E-04 – 7.45E-22
Cell death and survival	205	1.85E-04 – 5.86E-19
Cellular development	202	1.85E-04 – 3.70E-14
Cellular growth and proliferation	201	1.85E-04 – 3.70E-14
Cell morphology	95	1.62E-04 – 2.98E-13
**Top upstream regulators**		
**Upstream regulators**	**Predicted activation**	**p-values**
TNF	Inhibited	1.36E-14
SP1	Inhibited	4.36E-11
SMARCA4	Inhibited	1.07E-09
Cg	Inhibited	4.57E-09
ATF4	Inhibited	5.51E-09
